# Potentiating the Efficacy of mRNA Vaccines through NIR‐II Imaging‐Guided Precise Vaccination

**DOI:** 10.1002/advs.202413014

**Published:** 2025-07-18

**Authors:** Mengfei Li, Xue Zheng, Xinyang Yu, Shaolong Qi, Shoujun Zhu, Guocan Yu, Songling Zhang

**Affiliations:** ^1^ Department of Obstetrics and Gynecology First Hospital of Jilin University Changchun 130021 P. R. China; ^2^ Joint Laboratory of Opto‐Functional Theranostics in Medicine and Chemistry The First Hospital of Jilin University Changchun 130021 P. R. China; ^3^ Key Laboratory of Bioorganic Phosphorus Chemistry & Chemical Biology Department of Chemistry Tsinghua University Beijing 100084 P. R. China

**Keywords:** cancer immunotherapy, lipid nanoparticles, mRNA vaccines, NIR‐II imaging, self‐assembly

## Abstract

mRNA vaccines have been widely implemented and revolutionized the landscape of oncology and epidemiology. Potent vaccination demands precise control over injection depth to ensure optimal lymphatic drainage, driving the development of non‐invasive imaging techniques to visualize the distribution and efficacy of mRNA vaccines. Herein, near‐infrared‐II (NIR‐II) lipid nanoparticles (NIR‐II‐LNPs) are fabricated for mRNA delivery by integrating a cationic fluorophore amphiphile (IR780‐C16) into traditional LNPs, enabling longitudinal, quantitative, and non‐invasive trafficking of mRNA vaccines. The in vivo behaviors of mRNA vaccines are characterized with different vaccination protocols in mice and rabbits by NIR‐II fluorescence imaging. Results proved that following a 25 µL injection of mRNA vaccines into the mouse thigh at a depth of 4 mm or a 500 µL injection volume into a rabbit at a depth of 7 mm via the intramuscular route, the vaccines rapidly and abundantly accumulated in the inguinal lymph nodes. Flow cytometry and tissue immunofluorescence analyses further validated that optimized vaccination parameters effectively activated antitumor immunity and greatly boosted the therapeutic efficacy in B16‐OVA and HPV‐associated mouse tumor models. NIR‐II‐LNPs offer a unique modality for non‐invasively monitoring the fate of vaccines, reinforcing its role in preclinical and clinical mRNA therapeutics.

## Graphical abstract

A supramolecular strategy is utilized to integrate IR780‐C16 lipids into conventional LNPs, pioneering a novel NIR‐II‐LNPs platform that elucidates the correlations between mRNA vaccines efficacy and vaccination parameters in a non‐invasive approach. Notably, optimal vaccines enrichment in the LNs and maximum anti‐tumor immune efficacy can only be achieved through precise injection at an appropriate depth. This work pioneers an innovative approach to potentiate the efficacy of mRNA vaccines through NIR‐II imaging‐guided precision vaccination.

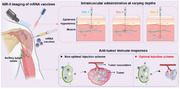

## 1 Introduction

mRNA vaccines based on lipid nanoparticles (LNPs) have emerged as a revolutionary strategy for cancer treatment and infectious disease prevention.^[^ ^1^, ^2^, ^3^, ^4^, ^5^, ^6^ ^]^ Therapeutic mRNA vaccines effectively trigger adaptive immunity by being timely delivered to secondary lymphoid tissues and expressing desired antigen proteins in the target cells.^[^ ^7^, ^8^, ^9^ ^]^ Precise vaccination is a prerequisite for mRNA vaccines to enhance their efficacy and minimize the risk of adverse events,^[^ ^10^, ^11^ ^]^ however, critical insights into the correlation between vaccination parameters and vaccines potency remain underexplored due to the lack of optimal imaging tools. There is an urgent need for a non‐invasive systemic imaging approach capable of correlating vaccines trafficking events, downstream adaptive immune responses with vaccination parameters. In the context of immuno‐oncology and emerging outbreaks, such a visual tool is favorable to boost the vaccines efficacy, streamline time, and offer substantial long‐term promise in advancing preclinical and translational vaccines research.

Molecular imaging offers non‐invasive, real‐time techniques for longitudinal monitoring of administrated vaccines, including positron emission tomography,^[^ ^12^ ^]^ computed tomography,^[^ ^13^ ^]^ magnetic resonance imaging,^[^ ^14^, ^15^ ^]^ and fluorescence imaging.^[^ ^16^, ^17^, ^18^ ^]^ Among various imaging modalities, NIR‐II fluorescence imaging offers unique opportunities for dynamically visualizing vaccines with unprecedented spatiotemporal resolution.^[^ ^19^, ^20^, ^21^ ^]^ IR780‐C16 iodide, an amphiphilic cationic heptamethine dye with considerable fluorescence intensity in the NIR‐II spectral region, is an excellent candidate for real‐time monitoring of mRNA vaccines and tracking immune response in vivo.^[^ ^22^, ^23^ ^]^ The satisfactory biosafety, self‐assembly ability, and charge characteristics of IR780‐C16 facilitate its integration into LNPs. The cationic sites in the heptamethine head facilitate mRNA complexation through electrostatic interactions. Additionally, the two hydrophobic alkyl tails confer superior co‐assembly capability with conventional LNPs components via hydrophobic interactions.

**Scheme 1:**
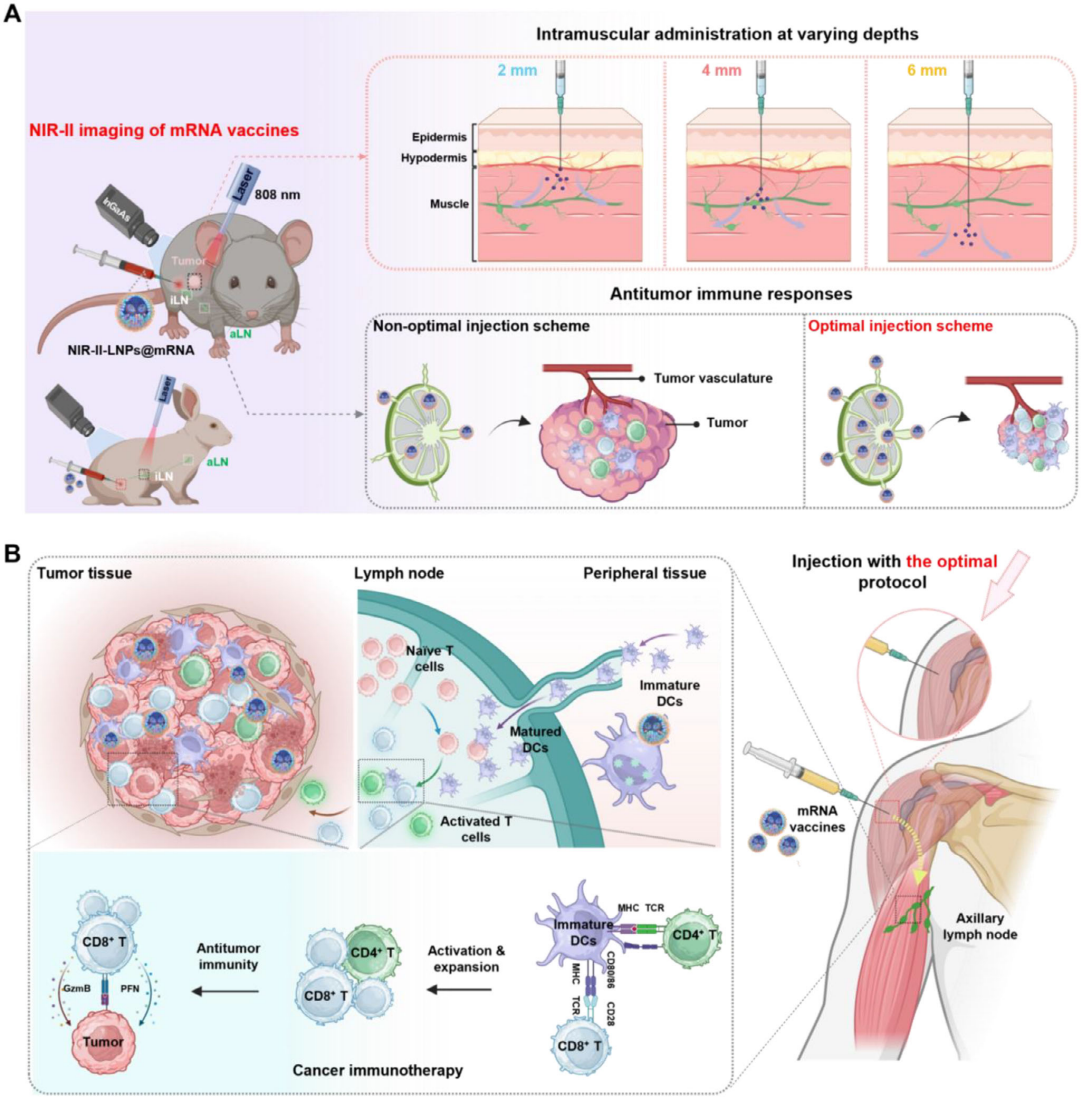
A) Schematic illustration of the correlation between vaccination parameters with vaccines distribution and potency. B) Schematic illustration of the antitumor mechanism of NIR‐II‐LNPs@mRNA at the optimal vaccination depth. Created with BioRender.com.

Herein, we develop novel NIR‐II fluorescent LNPs (NIR‐II‐LNPs) by integrating IR780‐C16 into LNPs to monitor vaccines distribution and verify the correlation between vaccination protocols and immune responses (**Scheme** **1**). Following vaccination at varying depths or volumes in mice and rabbits, NIR‐II‐LNPs allow non‐invasive, dynamic, and quantitative tracking of the migration and uptake of mRNA vaccines. Notably, mRNA vaccines swiftly migrated to inguinal lymph nodes (iLNs) following optimal‐depth intramuscular injection, whereas non‐optimal protocols resulted in a prolonged signal retention at the injection site. We established mRNA cancer vaccines (NIR‐II‐LNPs@mRNA^OVA^, NIR‐II‐LNPs@mRNA^E7^) to further analyze the influence of their dynamics on immune responses and antitumor efficacy in B16‐OVA and HPV‐associated tumor‐bearing mice. Immunization of NIR‐II‐LNPs@mRNA with optimized vaccination conditions markedly enhanced dendritic cells (DCs) maturation, elevated antigen‐specific T lymphocyte levels, and enhanced the antitumor performance. This research underscores the exceptional potential of NIR‐II‐LNPs as next generation carriers for optimizing mRNA vaccines vaccination and enhancing vaccines efficacy.

## 2 Results and Discussion

**Figure 1:**
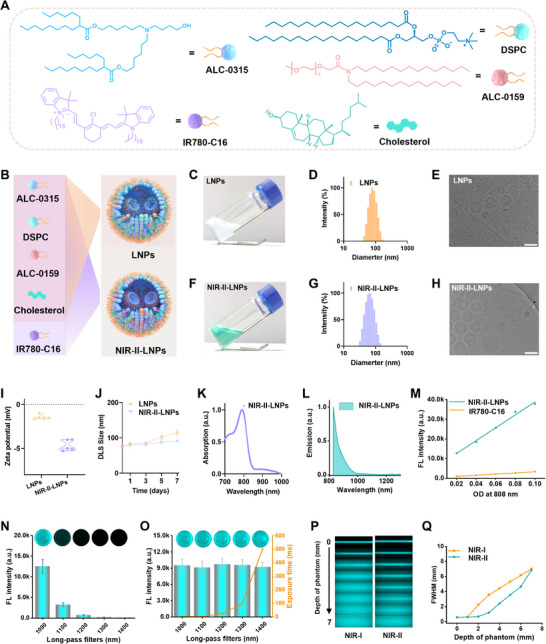
A) Chemical structures and schematic diagrams of ALC‐0315, DSPC, ALC‐0159, IR780‐C16, and cholesterol. B) Schematic illustration of LNPs and NIR‐II‐LNPs. C, F) Photographs of LNPs and NIR‐II‐LNPs solutions. D, G) DLS results of LNPs and NIR‐II‐LNPs. E, H) Cryo‐TEM images of LNPs and NIR‐II‐LNPs. Scale bars: 100 nm. I) Zeta potential analyses of LNPs and NIR‐II‐LNPs. J) Stability evaluation of LNPs and NIR‐II‐LNPs in PBS over 7 days by DLS. K, L) Absorption and emission spectra of NIR‐II‐LNPs in PBS. M) Integrated fluorescence intensity of NIR‐II‐LNPs and IR780‐C16 dye was plotted against OD value at 808 nm. FL intensity of NIR‐II‐LNPs at varying wavelengths under 75 mW cm^−2^ laser irradiation for 5 ms N) and with increasing exposure times O). P) Fluorescence images of capillaries loaded with NIR‐II‐LNPs submerged in 1% intralipid at varied depth in different detection windows. Q) FWHM of gaussian‐fitted intensity data in P) as a function of intralipid depth.

IR780‐C16 was specially designed and synthesized according to the procedure outlined in the experimental section (Figure S1, Supporting Information).^[^ ^24^, ^25^, ^26^ ^] 1^H NMR and liquid chromatograph mass spectrometer confirmed the successful synthesis and its high purity (Figures S2–S7, Supporting Information). IR780‐C16 was employed to label conventional LNPs for NIR‐II fluorescence imaging with millimetre‐scale penetration depth and excellent signal‐to‐background ratio.^[^ ^27^, ^28^ ^]^ The LNPs comprised [(4‐hydroxybutyl) azanediyl] bis (hexane‐6,1‐diyl) bis (2‐hexyldecanoate) (ALC‐0315), 1,2‐distearoyl‐sn‐glycero‐3‐phospho‐choline (DSPC), polyethylene glycol lipids (ALC‐0159), and cholesterol at molar ratios of 50%, 10%, 1.5%, and 38.5%, respectively (**Figure** **1**A–C). For the NIR‐II‐LNPs, IR780‐C16 was integrated at 0.5% of the total lipid weight, while the proportions of the other components remained unchanged (Figure 1A,B). The IR780‐C16 encapsulation efficiencies for NIR‐II‐LNPs were higher than 96% (Figure S8, Supporting Information). The physicochemical properties of LNPs and NIR‐II‐LNPs were investigated, dynamic light scattering (DLS) measurements indicated that the hydrodynamic diameters of LNPs and NIR‐II‐LNPs were 78.4 ± 1.7 nm and 75.0 ± 1.6 nm, respectively (Figure 1D,G). Cryo‐transmission electron microscopy (Cryo‐TEM) revealed that both LNPs and NIR‐II‐LNPs exhibited uniform spherical morphology (Figure 1E,H). Additionally, the zeta potentials of LNPs and NIR‐II‐LNPs were comparable, suggesting that the incorporation of IR780‐C16 merely affected the co‐assembly (Figure 1I). It should be noted that the existence of IR780‐C16 did not attenuate the stability, and negligible changes in diameter were observed after one week incubation in PBS (Figure 1J).

UV‐vis spectrum revealed a maximum absorption peak at 790 nm for the NIR‐II‐LNPs, accompanied by a noticeable color change upon formation (Figure 1C,F,K). NIR‐II‐LNPs exhibited bright emission in the state‐of‐the‐art NIR‐II spectral region (Figure 1L). Interestingly, the incorporation of IR780‐C16 within NIR‐II‐LNPs promoted the quantum yields (QYs), which were 11.3 times higher than that of free IR780‐C16 (Figure 1M; Figure S9, Supporting Information). The improved QYs were attributed to suppressed π‐π stacking and restricted internal rotation of IR780‐C16 within NIR‐II‐LNPs, minimizing aggregation‐induced quenching.^[^ ^29^ ^]^ Moreover, the alkyl chains increased hydrophobicity and reduced the interaction with surrounding water, optimizing their NIR‐II imaging performance.^[^ ^30^ ^]^ Based on the aforementioned factors, NIR‐II‐LNPs displayed a comparable fluorescence (FL) intensity at even 1400‐nm sub‐NIR‐II window under continuous irradiation with 808 nm light (Figure 1N,O). Penetration depths and spatial resolution in NIR‐I (850–900 nm) and NIR‐II (>1100 nm) windows were evaluated using 1% intralipid as a tissue mimic. Capillary visibility diminished with increasing tissue depth (0–7 mm) for both windows. NIR‐I imaging exhibited limited penetration (<3 mm) and severe scattering, whereas NIR‐II imaging (>1100 nm) revealed distinct capillary edges at 7 mm (Figure 1P). Correspondingly, NIR‐II fluorescence imaging offered superior capillary resolution with lower full widths at half maximum (FWHM) (Figure 1Q). These results highlighted the suitability of NIR‐II‐LNPs for deep tissue imaging.

**Figure 2:**
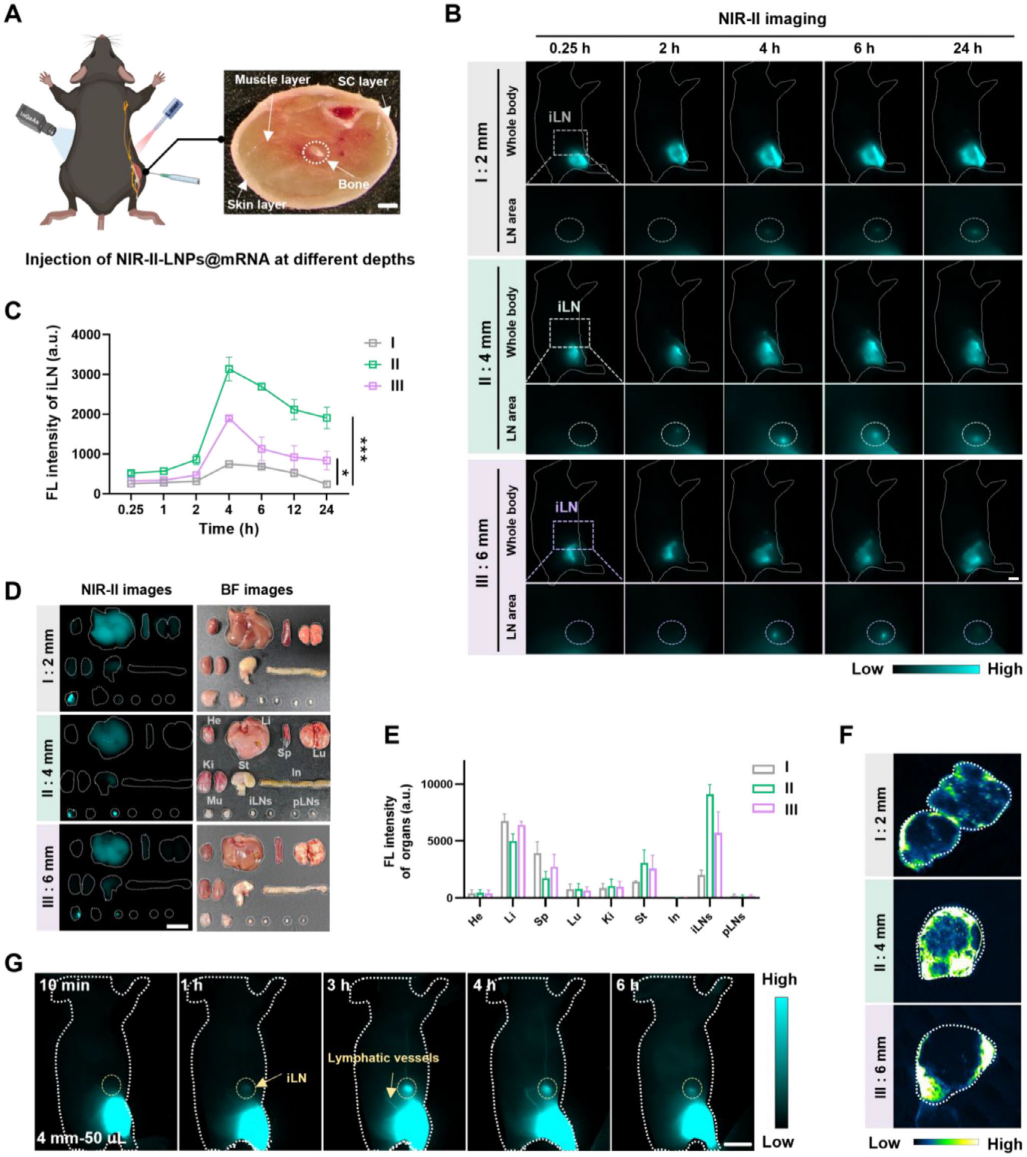
A) A schematic of the NIR‐II imaging study with C57BL/6J mice receiving different injection protocols. Photograph of the transverse section area of the mice hindlimb, highlighting fat, muscle thickness, and injection site. Scale bar: 1 mm. B) Whole‐body and high‐magnification NIR‐II fluorescent images of the mice administrated with NIR‐II‐LNPs@mRNA. Scale bars = 1 cm. C) Time‐dependent changes of FL intensity in the iLNs. Data are presented as the mean ± s.d. (*n* = 3). Statistical significance was determined by one‐way ANOVA with Tukey's multiple comparisons. *p < 0.05, **p < 0.01, ***p < 0.001. D) EX vivo fluorescence imaging of muscle, LNs, and main organs harvested from the mice at 24 h post‐injection. He, heart; Li, liver; Sp, spleen; Lu, lung; Ki, kidney; St, stomach; In, intestine; Mu, muscle; iLNs, inguinal lymph nodes; pLNs, popliteal lymph nodes. E) Quantification of FL intensity in excised muscle, LNs, and main organs. F) Whole‐slide imaging analysis of vaccines distribution. G) Real‐time fluorescence imaging of mRNA vaccines after intramuscular injection of NIR‐II‐LNPs@mRNA with the optimal protocol.

Intramuscular injection is a widely adopted vaccination method, in which the injection depth plays a pivotal role in activating immune responses.^[^ ^31^, ^32^ ^]^ However, rare systemic and non‐invasive studies have explored the correlation between vaccination depth and efficacy to provide guidance for optimal vaccination. Encouraged by the promising results in NIR‐II fluorescence imaging, we employed NIR‐II‐LNPs as delivery vectors to visualize the tracking and biodistribution of mRNA vaccines. We administered 25 µL of NIR‐II‐LNPs@mRNA in the right thigh of C57BL/6 mice at varying depths (Group I: 2 mm; Group II: 4 mm; Group III: 6 mm) to assess the in vivo lymphatic drainage and systemic biodistribution of mRNA vaccines (**Figure** **2**A). Whole‐body and inguinal lymph nodes (iLNs) NIR‐II fluorescence imaging was performed at different time points post‐injection, revealing the distribution of NIR‐II‐LNPs@mRNA over time (Figure 2B). For the mice in group I, the signals in the iLNs regions gradually increased and attained maxima at 4 hours post‐injection of NIR‐II‐LNPs@mRNA vaccines, with intergroup brightness differing despite uniform dosing (Figure 2C). In contrast, group II and group III exhibited substantial accumulation in the iLNs over 24 h post‐injection, with the fluorescence intensity in group II markedly exceeding that of group I and group III. Mice were euthanized at 24 h post‐injection and the main tissues (muscle, LNs, and major organs) were harvested to evaluate the biodistribution and clearance of the vaccines. It was apparent that vaccines predominantly localized in the injection site, liver, spleen, and iLNs. Faint or undetectable signals were observed in the iLNs of mice from group I and group III, whereas the mice in group II showed much higher signals, aligning with the in vivo imaging observations (Figure 2D,E). Subsequently, we conducted histological analysis of *ex vivo* iLNs to evaluate the spatial distribution of the vaccines. The fluorescence imaging of iLNs sections showed that the highest accumulation of NIR‐II‐LNPs was observed in group II, which was 4.4‐ and 2.0‐fold higher than the mice in group I and group III (Figure 2F; Figure S10, Supporting Information). These data confirmed a strong correlation between injection depth and signal intensity in the iLNs.

Given the limited interstitial volume from the vaccination site to iLNs, it is hypothesized that the volume of the vaccines formulation may influence its dynamics. To validate this conjecture, intravital iLNs imaging was conducted immediately after administering 5 µg of NIR‐II‐LNPs@mRNA vaccines at the optimal depth but with varying injection volumes. As shown in Figure S11A (Supporting Information), C57BL/6J mice were intramuscularly injected in the right thigh with volumes of 25 µL, 50 µL, and 100 µL, respectively (Group A: 25 µL; Group B: 50 µL; Group C: 100 µL). The dynamic migration of mRNA vaccines in iLNs and liver was monitored by NIR‐II fluorescence imaging at predetermined time points (Figure S11B,C, Supporting Information). Notably, volumes of 50 and 100 µL were sufficient to promptly deliver the vaccines to the axillary LNs (aLNs), whereas no signal was observed in the aLNs for 25 µL‐treated mice. Mice treated with 50 µL NIR‐II‐LNPs@mRNA showed significantly higher FL intensity in the iLNs compared to other groups, suggesting a more effective translocation of vaccines from the injection site to the iLNs (Figure S11D, Supporting Information). Subsequently, the injected muscle, iLNs, popliteal lymph nodes (pLNs), and major organs were isolated, with contralateral tissues collected as controls. FL intensity was measured to quantify the distribution of mRNA vaccines. Although vaccines accumulation in the iLNs was comparable following administration of 50 µL and 100 µL formulations, the latter exhibited more pronounced signals in the liver (Figure S11E–G, Supporting Information). These findings verified that the kinetics of vaccines transport to LNs were influenced by the volume of vaccines formulation. The results of real‐time fluorescence imaging illustrated that the NIR‐II‐LNPs@mRNA vaccines traveled through lymphatic vessels from the injection site to the iLNs and aLNs under optimal vaccination conditions (Figure 2G; Figure S12, Supporting Information).

**Figure 3:**
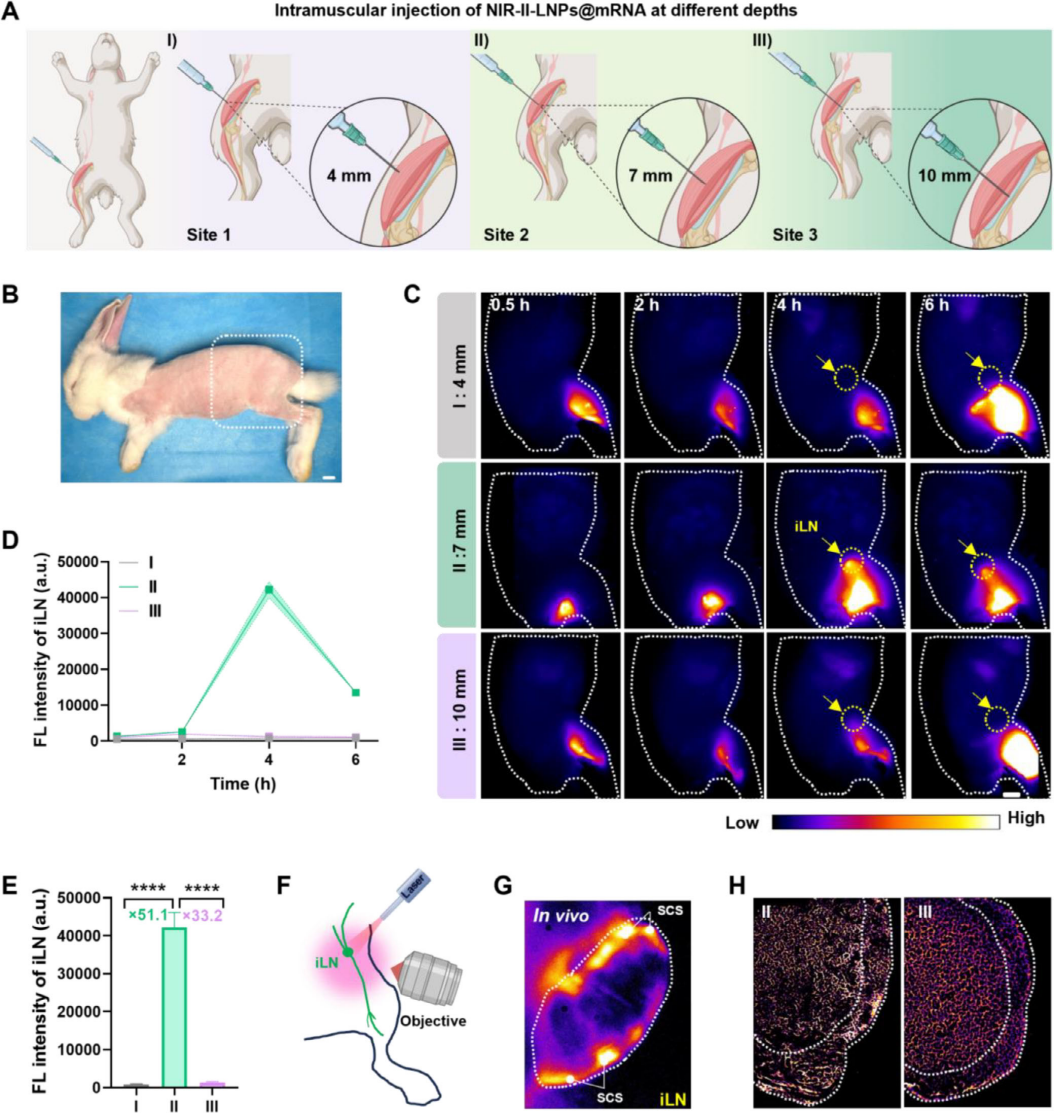
A) Schematic illustration of the injection regimens. B) The white dotted box in the photograph indicates the region scanned by NIR‐II fluorescence imaging. C) In vivo imaging of iLNs at different time points (scale bar  =  1 cm). D) Quantification of FL intensity in the iLNs over time. E) Comparison of FL intensity of iLNs from different treatments. F) Schematic illustration of in vivo study utilizing NIR‐II zoom‐stereo microscopy. G) In vivo high‐resolution NIR‐II fluorescence imaging of iLNs. The arrows depict the SCS in the periphery of iLNs. H) Histological analysis of vaccines uptake by APCs in the draining LNs. Data are presented as the mean ± s.d. (*n* = 3). *p < 0.05, **p < 0.01, ***p < 0.001, ****p < 0.0001. I, 4 mm‐500 µL; II, 7 mm‐500 µL; III, 10 mm‐500 µL.

To exclude species‐specific differences in vaccines biodistribution that could impede clinical translation, the biodistribution of NIR‐II‐LNPs@mRNA was evaluated in female rabbits. Rabbits were intramuscularly injected with 500 µL of NIR‐II‐LNPs@mRNA at three different depths (**Figure** **3**A,B). NIR‐II‐LNPs@mRNA rapidly accumulated in the iLNs of 7 mm‐treated rabbits, whereas negligible or no signals were detected in the iLNs of 4 mm‐ and 10 mm‐injected rabbits within 4 h post‐injection (Figure 3C–E). It was evident that effective vaccines delivery depended on precise administration at the ideal depth, thereby validating the accuracy and generalizability of the aforementioned findings. To further elucidate vaccines distribution in the iLNs, we utilized NIR‐II zoom‐stereo microscopy to meticulously examine lymphatic uptake within the iLNs (Figure 3F,G). As depicted in Figure 3H, the fluorescence signal of NIR‐II‐LNPs@mRNA predominantly localized in the subcapsular sinus (SCS) at the periphery of the iLNs, indicating that antigens arrived through afferent lymphatic vessels and were captured by antigen‐presenting cells (APCs) along the SCS. In general, NIR‐II‐LNPs@mRNA integrated NIR‐II fluorescence imaging with nanovaccines, facilitating real‐time tracking of their migration and retention in iLNs. This innovative approach offers a robust tool for optimizing vaccines dosage and injection protocols.

**Figure 4:**
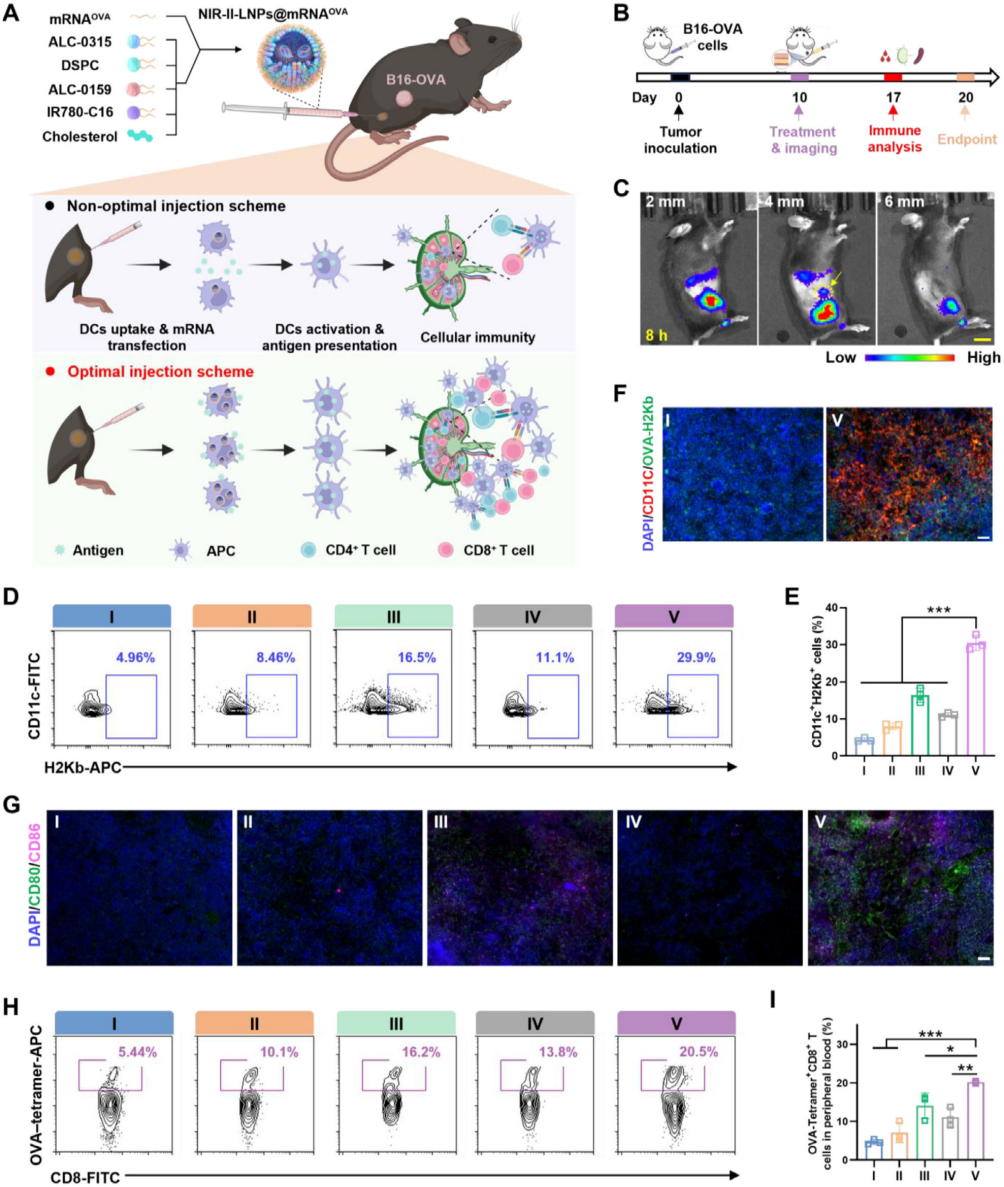
A) Vaccines administered with optimal schedules enhance APC maturation, facilitate APC‐T cell interactions, and stimulate the production of multifunctional T cells and antibodies. This strategy ensures durable immunity while significantly conserving antigen doses. B) Therapeutic schedule depicting vaccination of B16‐OVA tumor‐bearing mice. C) In vivo bioluminescence imaging 8 h after intramuscular injections of NIR‐II‐LNPs@mRNA^OVA‐Luc^ at varying depths (*n* = 3). D, E) Representative flow plots D) and quantitative analysis E) of CD11c^+^H2Kb^+^ DCs in dLNs from mice treated with different strategies. F) Representative immunofluorescence staining of OVA‐H2Kb on DCs in dLNs for control (I) and 4 mm‐50 µL (V) treatment groups. Scale bar, 20 µm. G) Immunofluorescence staining of DCs maturation in dLNs. H) FCM results and I) quantitative analysis of OVA‐tetramer^+^CD8^+^ T cells in peripheral blood after different treatments. Data are presented as the mean ± s.d. (*n* = 3). *p < 0.05, **p < 0.01, ***p < 0.001. I, control; II, 2 mm‐25 µL; III, 4 mm‐25 µL; IV, 6 mm‐25 µL; and V, 4 mm‐50 µL.

LNs serve as crucial sites where APCs interact with naïve T cells to elicit T‐cell‐dependent cellular immunity. An ideal vaccination regimen should enhance APCs maturation, boost APCs‐T cell interactions, and stimulate potent immune responses, thereby achieving long‐lasting immunity with reduced antigen usage (**Figure** **4**A). We evaluated and compared the in vivo immune responses in unilateral B16‐OVA tumor‐bearing C57BL/6 mice following vaccination with different regimens, by examining APCs in the tumor‐draining lymph nodes (dLNs) and T cells in both the dLNs and peripheral blood (Figure 4B). Initially, 5 µg of OVA (257‐264)‐luciferase‐encoding mRNA (mRNA^OVA‐Luc^) was encapsulated in NIR‐II‐LNPs (final volume: 25 µL) and administered intramuscularly into the right thigh of mice at depths of 2 mm, 4 mm, and 6 mm, respectively. Aligned with the NIR‐II imaging outcomes, dLNs exhibited the highest luminescence following intramuscular injection at a depth of 4 mm, offering compelling evidence of enhanced translation in vivo (Figure 4C; Figure S13, Supporting Information). These results suggested that an appropriate injection depth enhanced vaccines retention in the dLNs and improved mRNA transfection efficiency. Antigen cross‐presentation by DCs is vital for triggering adaptive immunity. To determine whether the vaccination parameters affected the antigen presentation induced by NIR‐II‐LNPs@mRNA^OVA^, we examined the expression of H‐2Kb/SIINFEKL on the surface of DCs by flow cytometry (FCM) or immunofluorescence staining. Specifically, the mice were divided into five groups based on vaccination depth and volume: control (I), 2 mm‐25 µL (II), 4 mm‐25 µL(III), 6 mm‐25 µL(IV), and 4 mm‐50 µL (V). Notably, vaccination with the 4 mm‐50 µL (V) regimen yielded a markedly higher proportion of H‐2Kb/SIINFEKL‐positive DCs (29.9%) compared to the control group (4.96%), confirming the robust immune activation triggered by NIR‐II‐LNPs@mRNA^OVA^ under optimized vaccination conditions (Figure 4D,E; Figure S14, Supporting Information). Immunofluorescence analysis consistently demonstrated immunization of NIR‐II‐LNPs@mRNA^OVA^ with optimized vaccination conditions induced the highest levels of H‐2Kb/SIINFEKL‐positive DCs, in comparison to the control group (Figure 4F). Given the excellent translation efficiency and antigen presentation, DCs activation was further verified by immunofluorescence staining. As depicted in Figure 4G and 4 mm‐50 µL group significantly elevated the population of CD80^+^CD86^+^ DCs in the dLNs in sharp contrast to the other groups. Inducing cytotoxic T lymphocytes (CTLs) against tumor‐specific antigens underpins vaccines‐mediated tumor eradication.^[^ ^33^, ^34^, ^35^ ^]^ Seven days after the second vaccination, antigen‐specific CD8^+^ T cells in the peripheral blood were analyzed using H‐2Kb/SIINFEKL tetramer staining. Compared to the control group (5.44%), the proportion of tumor‐specific T cells markedly increased to 10.1%, 16.2%, 13.8%, and 20.5% in mice vaccinated with 2 mm‐25 µL, 4 mm‐25 µL, 6 mm‐25 µL, and 4 mm‐50 µL, respectively (Figure 4H,I; Figure S15, Supporting Information). Additionally, to determine the impact of vaccination on antigen‐specific immunity, we quantified antigen‐specific antibody titers in mouse sera under different treatment regimens.^[^ ^36^ ^]^ As depicted in Figure S16 (Supporting Information), the NIR‐II‐LNPs@mRNA^OVA^ vaccine elicited a significant elevation in OVA‐specific immunoglobulin G (IgG) antibody titers targeting the B16F10‐OVA tumor antigen, especially under the optimized vaccination parameters. Together, these data suggested that therapeutic efficacy of NIR‐II‐LNPs@mRNA^OVA^ vaccine was significantly improved through the optimization of vaccination parameters, with negligible changes observed in body weight (Figure S17, Supporting Information).

**Figure 5:**
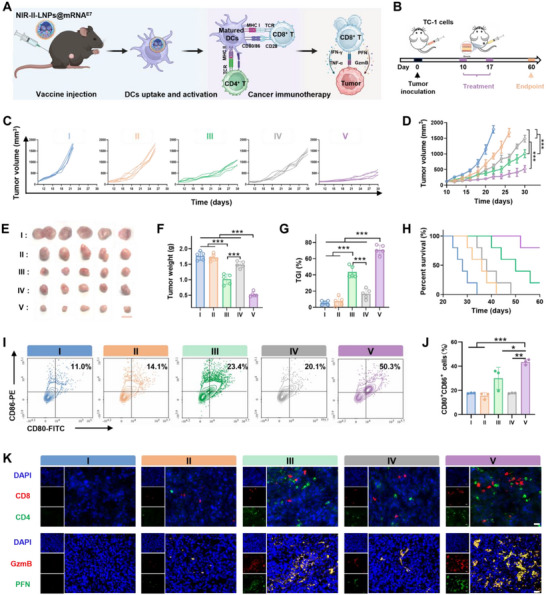
A) The therapeutic mechanism of the NIR‐II‐LNPs@mRNA^E7^ cancer vaccines. B) The schedule of TC‐1 subcutaneous tumor establishment and mRNA vaccination. C) Individual and D) average tumor growth curves of the mice received indicated treatments. E) Representative images of TC‐1 tumors of mice treated as indicated. Scale bar: 1 cm. F) Final tumor weights of the tumor‐bearing mice after different treatments. G) TGI rate of the mice in each group. H) Survival curves of the HPV‐associated tumor‐bearing mice after different treatments. I) FCM plots and J) quantitative frequency of CD80^+^CD86^+^ cells in dLNs after different treatments. K) Representative immunofluorescence images of intratumoral CD4^+^CD8^+^ T cells and GzmB/PFN secretion in indicated groups. Scale bar: 20 µm. *p<0.05; **p < 0.01; ***p < 0.001. I, PBS; II, 2 mm‐25 µL; III,4 mm‐25 µL; IV, 6 mm‐25 µL; and V, 4 mm‐50 µL NIR‐II‐LNPs@mRNA^E7^ treatments.

Subsequently, we evaluated the antitumor efficacy of NIR‐II‐LNPs@mRNA^E7^ vaccines in a TC‐1 subcutaneous tumor model. Therapeutic vaccines targeting the E7 oncoprotein are effective in combating high‐risk HPV strains, particularly HPV16, which is a major contributor to cervical cancer.^[^ ^37^, ^38^, ^39^ ^]^ Mice were inoculated with TC‐1 cells on day 0, followed by immunization with a prime vaccines on day 10 and a booster vaccines on day 17 (**Figure** **5**A,B). The antitumor efficacy of the NIR‐II‐LNPs@mRNA^E7^ vaccines was evaluated by tracing the tumor growth profile (Figure 5C,D). Impressively, employing the appropriate vaccination parameters (4 mm‐50 µL) resulted in sustained tumor suppression, yielding a remarkable 70.9% tumor growth inhibition (TGI) efficiency for NIR‐II‐LNPs@mRNA^E7^ (Figure 5E–G and Table S1). Correspondingly, the enhanced antitumor immune response significantly prolonged the median survival rate of mice vaccinated with NIR‐II‐LNPs@mRNA^E7^ (Figure 5H). To further elucidate the mechanism underlying the observed reduction in tumor growth, we sacrificed all immunized mice to harvest dLNs and tumors to analyze tumor‐infiltrating immune cells via FCM and immunofluorescence. Compared with the control group (11.0%), mice treated with NIR‐II‐LNPs@mRNA^E7^ exhibited significantly higher average percentages of CD86^+^CD80^+^ cells, with values of 14.1% (II), 23.4% (III), 20.1%(IV), and 50.3%(V), respectively (Figure 5I,J). Notably, the 4 mm‐50 µL treatment group exhibited the highest percentage of CD80^+^CD86^+^ cells, indicating excellent DCs activation, which in turn fostered the proliferation of CTLs. To further substantiate this hypothesis, tumors were extracted and subjected to histological immunofluorescence analysis, as illustrated in Figure 5K. The immunofluorescence images revealed minimal CD8 and CD4 T cells infiltration in PBS‐treated tumors, contrasting with notably higher levels in the experimental group. Moreover, the administration of NIR‐II‐LNPs@mRNA^E7^ induced a massive amplification of cytotoxic molecules secreted by tumor‐killing CTLs, including perforin (PFN) and granzyme B (GzmB), which provided compelling evidence for the proposed mechanism and elucidated the vaccine's remarkable antitumor efficacy. Finally, we assessed the systemic toxicity of the vaccines by examining the standard blood indicators as well as liver and renal function (Figures S18 and S19, Supporting Information). No significant fluctuations were observed, and body weights remained basically unchanged across all groups (Figure S20, Supporting Information), suggesting that the NIR‐II‐LNPs‐based mRNA^E7^ vaccines were well tolerated without apparent toxicity. H&E staining of major organs revealed no tissue damage, further confirming the absence of acute toxicity or systemic inflammation (Figure S21, Supporting Information). The cumulative results indicated that vaccines could trigger T‐cell immunity irrespective of the administration route, but the quality of specific responses varies. Nevertheless, vaccines achieved maximal efficacy and minimized adverse events only when precisely localized to the tissues requiring intervention.

## 3 Conclusion

In summary, we successfully engineered novel NIR‐II luminescent LNPs, termed as NIR‐II‐LNPs, for highly efficient mRNA delivery and non‐invasive imaging of vaccines trafficking. The NIR‐II tracking data presented here directly demonstrate that the depth and volume of vaccines greatly impact the in vivo fate and, ultimately, modulate the magnitude of vaccine‐specific immune responses. Our experiments revealed that nanovaccines can migrate more efficiently to the iLNs via lymphatic vessels when precisely localized at the appropriate tissue depth. Arising from the high accumulation in LNs and the robust DCs activation under optimal vaccination parameters, the tumor vaccines (NIR‐II‐LNPs@mRNA^OVA^, NIR‐II‐LNPs@mRNA^E7^) exhibited exceptional antitumor performance in both B16‐OVA and HPV‐associated mouse tumor models. In contrast to prior studies conducted *ex vivo* following euthanasia of mice, our research pioneered a non‐invasive, longitudinal, and real‐time imaging strategy to track mRNA vaccines in vivo, thereby offering valuable insights into the exploration and validation of vaccines efficacy across diverse vaccination conditions. We anticipate that this promising platform can be adapted for other vaccines studies in vivo, facilitating quick immunity verification and designing more effective vaccines.

## 4 Experimental Section

### Materials

2,3,3‐Trimethyl‐3H‐indole, 1‐iodohexadecane, cyclohexanone, and phosphorus oxychloride were sourced from TCI. N, N‐dimethylformamide (DMF), toluene, dichloromethane (DCM), diethyl ether, and acetonitrile were obtained from Meryer and Aladdin. ALC‐0315, DSPC, and ALC‐0159 were purchased from XIAMEN SINOPEG BIOTECH CO., LTD. All commercial reagents were used without further purification. GFP‐coding RNA (5mo‐U) was acquired from APExBIO. The T7 High Yield RNA Transcription Kit, Vaccinia Capping System, and Cap 2′‐O‐Methyltransferase were supplied by Vazyme. E. coli Poly(A) Polymerase was provided by Novoprotein. OVA Tetramer‐SIINFEKL‐APC came from MBL, OVA 257−264 peptide from Genscript, and Golgi stop reagent from BD. ^1^H NMR spectra were recorded on a JNM‐ECZ400S/L1. Anti‐CD11c‐FITC, Anti‐CD80‐PE, Anti‐CD86‐APC, anti‐CD4‐FITC, and anti‐CD8‐PE antibodies were sourced from BioLegend, Inc. (San Diego, USA), while anti‐CD3 and anti‐CD8 antibodies were obtained from Abcam (UK). A PerkinElmer Lambda 950 was employed to record ultraviolet‐visible‐near‐infrared (UV‐VIS‐NIR) absorption spectra, and fluorescent emission spectra were captured by an Edinburgh FL 920 spectrofluorometer. CLSM images were taken with an Olympus FV3000 microscope. The particle sizes of the LNPs were analyzed using a DLS analyzer (Zetasizer Nano ZS90, Malvern Instruments) at a 90° detection angle at 25 °C.

### Synthesis of Compound 1

Initially, phosphorus oxychloride (7.81 g, 51.0 mmol) in 5 mL DCM was added dropwise to a precooled solution of DMF (3.67 g, 3.85 mL, 50 mmol) in 5 mL DCM. After stirring for 30 min, cyclohexanone (2.5 g, 25.5 mmol) in 5 mL DCM was slowly introduced into the reaction mixture, which was then heated at 80 °C for 4 h. The above mixture was subsequently cooled to room temperature and poured into an ice‐water bath. The resulting mixture was left undisturbed overnight, resulting in a yellow solid powder.

### Synthesis of Compound 2

A mixture of 2,3,3‐trimethyl‐3H‐indole (1.0 g, 6.28 mmol) and 1‐iodohexadecane (6.64 g, 18.8 mmol) was stirred at 100 °C for 15 h in a 40 mL solvent blend of toluene and acetonitrile (1:1). After cooling to room temperature, the mixture was slowly added into ice‐cold diethyl ether, affording a red precipitate. The product was collected, washed with 30 mL of petroleum ether, and purified by column chromatography utilizing a gradient elution of dichloromethane/methanol mixture (30:1, v/v), yielding a pale purple solid powder.

### Synthesis of IR780‐C16

Compound 1 (0.23 g, 1.34 mmol) and compound 2 (0.84 g, 2.67 mmol) were dissolved in a 50 mL mixture of toluene and ethanol (1:1) in a round‐bottom flask. The solution was refluxed for 12 hours, producing a dark green solution. Upon completion, the solvent was evaporated under reduced pressure to obtain the crude product. The material was purified via silica gel column chromatography using a DCM and methanol mixture (20:1, v/v) as the eluent, ultimately resulting in a green solid powder.

### Synthesis and Characterization of NIR‐II‐LNPs

A lipid solution in ethanol was prepared by mixing ALC‐0315, DSPC, ALC‐0159, cholesterol, and IR780‐C16. IR780‐C16 was integrated at 0.5% of the total lipid weight. mRNA (e.g., mRNA^Luc^, mRNA^EGFP^, mRNA^OVA^, or mRNA^E7^) was diluted in 50 mM citrate buffer (pH 4.0, Fisher) to form the aqueous phase. The lipid and mRNA solutions were rapidly mixed through microfluidic devices (Micro&Nano Technologies) at a 3:1 aqueous‐to‐ethanol ratio by volume to manufacture NIR‐II‐LNPs. Followed by dialysis against PBS to remove the ethanol for in vivo experiments. The DLS size, zeta potential, and morphology of the NIR‐II‐LNPs were characterized using a Zetasizer Nano ZS90 (Malvern Panalytical, Malvern, UK) and cryo‐EM. The IR780‐C16 encapsulation efficiencies for NIR‐II‐LNPs were measured using the PerkinElmer Lambda 950. The encapsulation efficiency = 1 – free IR780‐C16 fraction.

### mRNA Synthesis

For mRNA coding the E7, luciferase and OVA (257–264) peptide, a cDNA template containing a T7 promoter, 5′ UTR, the coding sequence, and 3′ UTR was designed. The entire sequence was synthesized and transcribed by General Bio. The mRNA underwent further purification using a phenol‐chloroform extraction kit, and its final concentration was determined using a Thermofisher Scientific NanoDrop microdevice.

### Quantum Yields Test

Fluorescence intensity in the region above 1100 nm was recorded with an InGaAs camera (Teledyne Princeton Instruments, NIRvana 640) under 808 nm excitation. An 850‐nm short‐pass (SP) filter (ThorLabs) served as the excitation filter, while a 1100‐nm long‐pass (LP) filter (ThorLabs) was utilized as the emission filter. The optical density (OD) at 808 nm for all samples was maintained below 0.1. NIR‐II fluorescence intensities were assessed using the same 808 nm laser. QYs of the samples were calculated from five different concentrations with varying ODs at 808 nm. By measuring the ODs at 808 nm alongside the spectrally integrated fluorescence intensity, the QYs of a test sample were determined according to the following equation:

(1) $$\varphi_{\chi }\left(\gamma \right)=\varphi_{\mathrm{std}}\left(\gamma \right)\times \frac{F_{\chi }}{F_{\mathrm{std}}}\times \frac{A_{\mathrm{std}}\left(\gamma \right)}{A_{\chi }\left(\gamma \right)}\times {\left(\frac{\eta_{\chi }}{\eta_{\mathrm{std}}}\right)}^{2}$$

### NIR‐II Imaging

Prior to imaging, all mice were shaved and anesthetized with either isoflurane or chloral hydrate. Subsequently, they were positioned on the imaging table, with at least three mice serving as parallel controls for each imaging session. NIR‐I/NIR‐II images were captured using a two‐dimensional InGaAs array with an 808 nm laser at a power density of 75 mW cm^−^^2^. Emissions were typically collected through various LP and SP filters. The InGaAs camera, with adjustable exposure times, was employed to acquire images in the NIR‐II window. The transmission regions for the LP filters are as follows: FELH900 (912‐2150 nm), FELH1000 (1013‐2150 nm), FELH1100 (1114‐2150 nm), FELH1200 (1215‐2150 nm), FELH1300 (1316‐2150 nm), and FELH1400 (1417‐2150 nm). For simplicity, the NIR‐I and NIR‐II spectral regions were categorized into approximate wavelength ranges: 850–900 nm, >900 nm, >1000 nm, >1100 nm, >1200 nm, >1300 nm, and >1400 nm.

### Animals and Cells

Female C57BL/6J mice (6‐8 weeks old) were purchased from Beijing Vital River Laboratory Animal Technology and maintained in SPF conditions at the animal facility of Tsinghua University. All animal procedures were conducted in strict accordance with an animal protocol approved by the Tsinghua University Animal Care and Use Committee (protocol no. 21‐YGC1.G22‐1). B16‐OVA and TC‐1 cells were purchased from the American Type Culture Collection. The selected cell lines were cultured in DMEM complete medium with 1% P/S (penicillin‐streptomycin) and 10% fetal bovine serum (FBS) (v/v %) at 37 °C in a CO_2_ incubator.

### Pharmacokinetics and Biodistribution Studies

A distinct biodistribution study was performed in healthy mice that received a single intracranial injection of either NIR‐II‐LNPs or NIR‐II‐LNPs@mRNA. NIR‐II fluorescence imaging was carried out using a two‐dimensional InGaAs camera over extended periods (Imaging condition: excitation at 808 nm, ≈75 mW cm^−2^ power density, and 100 ms exposure time). Mice were euthanized 24 h post‐administration of NIR‐II‐LNPs or NIR‐II‐LNPs@mRNA. The IR780‐C16 content in the harvested tumor tissues and organs was analyzed via NIR‐II fluorescence imaging. Additionally, various formulations were injected into subcutaneous HPV tumor‐bearing mice, tumor tissues, and major organs were collected for H&E staining to evaluate in vivo toxicity.

### In Vivo Imaging of Luciferase Protein Expression

B16‐OVA‐Luc tumor‐bearing mice were immunized and subsequently injected intraperitoneally with 100 µL PBS containing 30 mg mL^−1^ d‐luciferin. Luminescence in vivo was monitored and quantified using the IVIS Lumina II charge‐coupled device imaging system.

### In Vivo Tumor Models and Treatment

Therapies commenced when tumors grew to 100 mm^3^. TC‐1 tumor‐bearing mice were divided into five groups, with each mouse receiving 5 µg of mRNA^E7^. Tumor sizes were measured by a digital caliper, and volume was calculated according to the following formula: volume = (width^2^ × length) / 2. TGI was calculated as: TGI = (1 − V_t_ /V_c_) × 100%, where V_t_ and V_c_ represent the relative tumor volumes of the treated and control groups, respectively.

### Flow Cytometric Analysis and Immunofluorescence Staining

Tumor or LNs suspensions were filtered through a 70 µm filter to obtain a single‐cell suspension. Cells were then stained with CD4, CD8, CD80, CD86, or CD11c (dilution ratio 1:100). The stained cells were washed with PBS and analyzed by flow cytometry. Data analysis was performed with FlowJo 10.0 software. Upon completion of the treatment schedule, mice were euthanized, and tumors or LNs were collected for immune analysis (CD8, CD4, granzyme B, perforin, CD11c, or OVA‐H2Kb) via immunofluorescence staining.

### Detection of OVA‐Specific IgG Antibody in Serum

The 48‐well High Bind Stripwell^TM^ plates (Corning) were precoated with 1 µg mL^−1^ of OVA‐IgG, followed by a blocking procedure with 1% BSA. The plates were then washed three times with wash buffer to remove unbound components. Serum samples were serially diluted in the blocking solution, added to the plates, and incubated for 2 h. Bound antibodies were detected using biotinylated anti‐OVA‐IgG at a concentration of 0.1 µg mL^−1^, and antibody binding was visualized with streptavidin‐HRP. The signal was developed with a TMB substrate kit, and absorbance was quantified at 450 nm using a SpectraMax™ 190 microplate reader.

### Statistical Analysis

Statistical analyses were conducted employing GraphPad Prism 9.5 with independent triplicate replication of all experiments to validate reproducibility. This rigorous analytical approach substantiated the robustness of findings. Data were expressed as mean ± standard deviation (SD). Comparisons between the two datasets were performed by the Student's t‐test, and statistical significance was defined as p < 0.05 (*p < 0.05, **p < 0.01, ***p < 0.001). Statistical significance of three or more groups was assessed using one‐way or two‐way ANOVA followed by Tukey's post hoc test.

## Conflict of Interest

The authors declare no conflict of interest.

## Supporting information



Supporting Information

## Data Availability

The data that support the findings of this study are available from the corresponding author upon reasonable request.
