# Supplementary material for: Potentiating the Efficacy of mRNA Vaccines through NIR‐II Imaging‐Guided Precise Vaccination
Source: Adv Sci (Weinh). 2025 Jul 18;12(37):e13014. doi: 10.1002/advs.202413014 (PMC12499424; doi:10.1002/advs.202413014)
Supplement: Supplementary file 1 — Supporting Information [file ADVS-12-e13014-s001.pdf]

ADVANCED SCIENCE
Open Access

Supporting Information

for *Adv. Sci.*, DOI 10.1002/advs.202413014

Potentiating the Efficacy of mRNA Vaccines through NIR-II Imaging-Guided Precise Vaccination

Mengfei Li, Xue Zheng, Xinyang Yu, Shaolong Qi, Shoujun Zhu, Guocan Yu* and Songling Zhang**

Supporting Information

Potentiating the Efficacy of mRNA Vaccines through NIR-II Imaging-Guided Precise Vaccination

Mengfei Li, Xue Zheng, Xinyang Yu, Shaolong Qi, Shoujun Zhu*, Guocan Yu* & Songling Zhang*

Dr. M. Li, Prof. S. Zhu, Prof. S. Zhang
Department of Obstetrics and Gynecology, First Hospital of Jilin University
Changchun 130021, P. R. China
E-mail: sjzhu@jlu.edu.cn, slzhang@jlu.edu.cn
Dr. M. Li, X. Zheng, Prof. S. Zhu, Prof. S. Zhang
Joint Laboratory of Opto-Functional Theranostics in Medicine and Chemistry, The First Hospital of Jilin University
Changchun, 130021, P. R. China
Dr. X. Yu, Dr. S. Qi, Prof. G. Yu
Key Laboratory of Bioorganic Phosphorus Chemistry & Chemical Biology, Department of Chemistry, Tsinghua University
Beijing 100084, P. R. China
E-mail: guocanyu@mail.tsinghua.edu.cn

WILEY-VCH

Supporting Information

Supplementary Figures

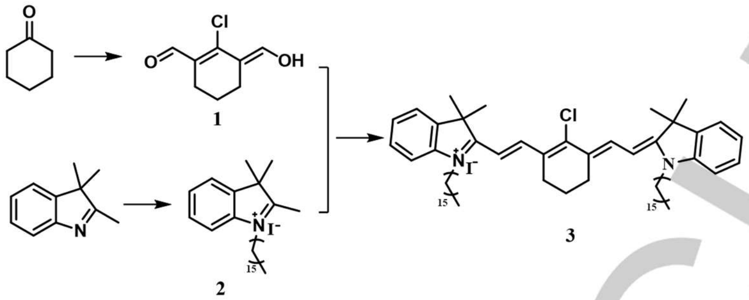

Figure S1. Synthetic route of IR780-C16.

WILEY-VCH

Supporting Information

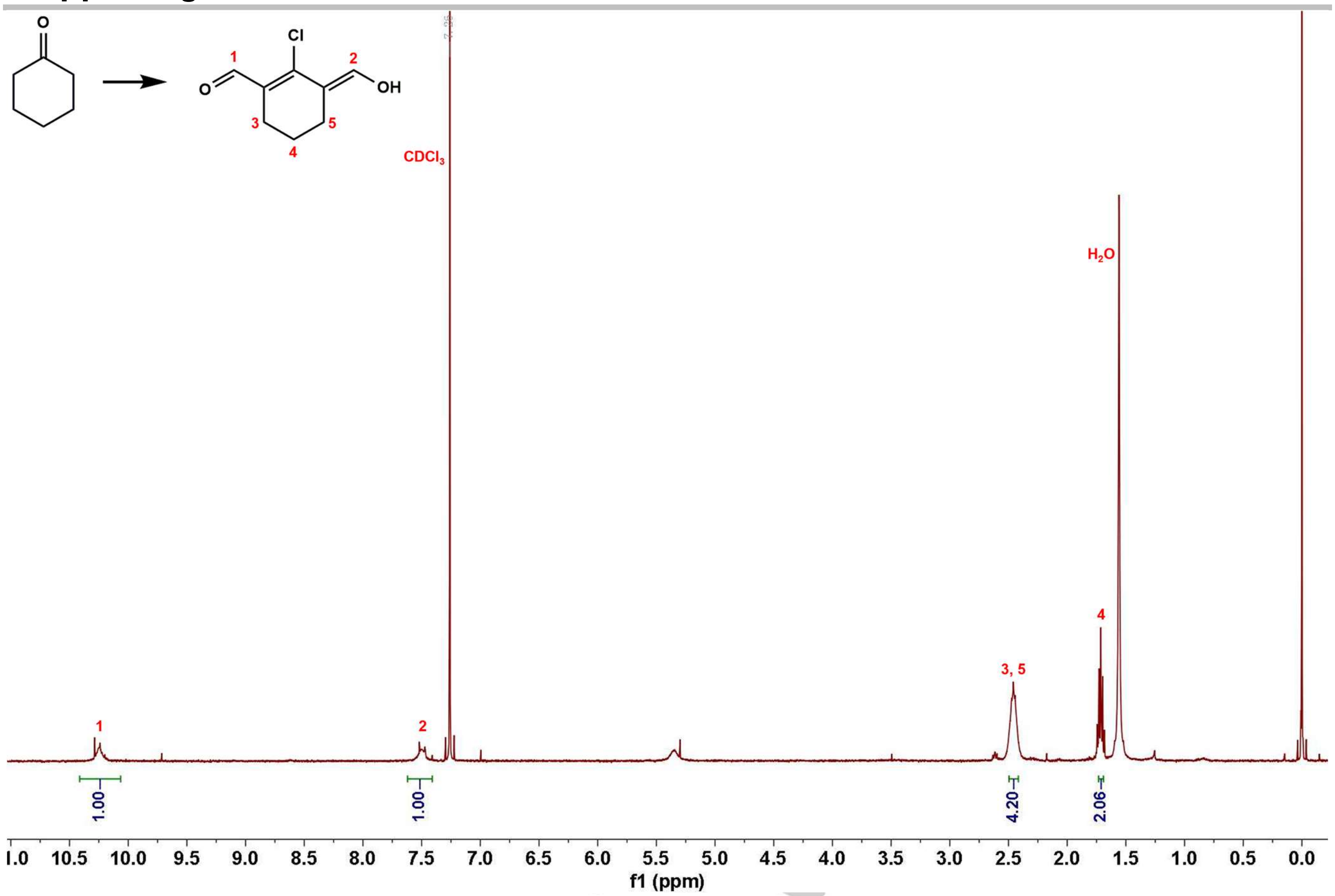

Figure S2. ^{1}H NMR spectrum of Compound 1 (400 MHz, 298K, $CDCl_3$).

WILEY

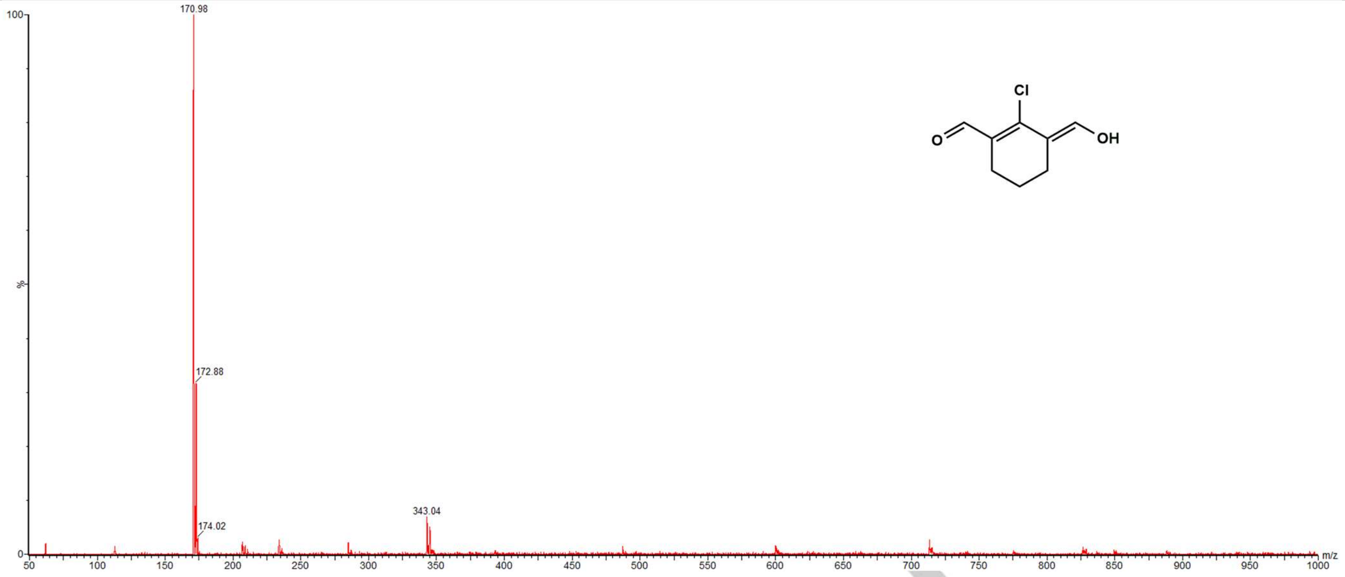

Figure S3. ESI-MS spectrum of Compound 1.

WILEY-VCH

Supporting Information

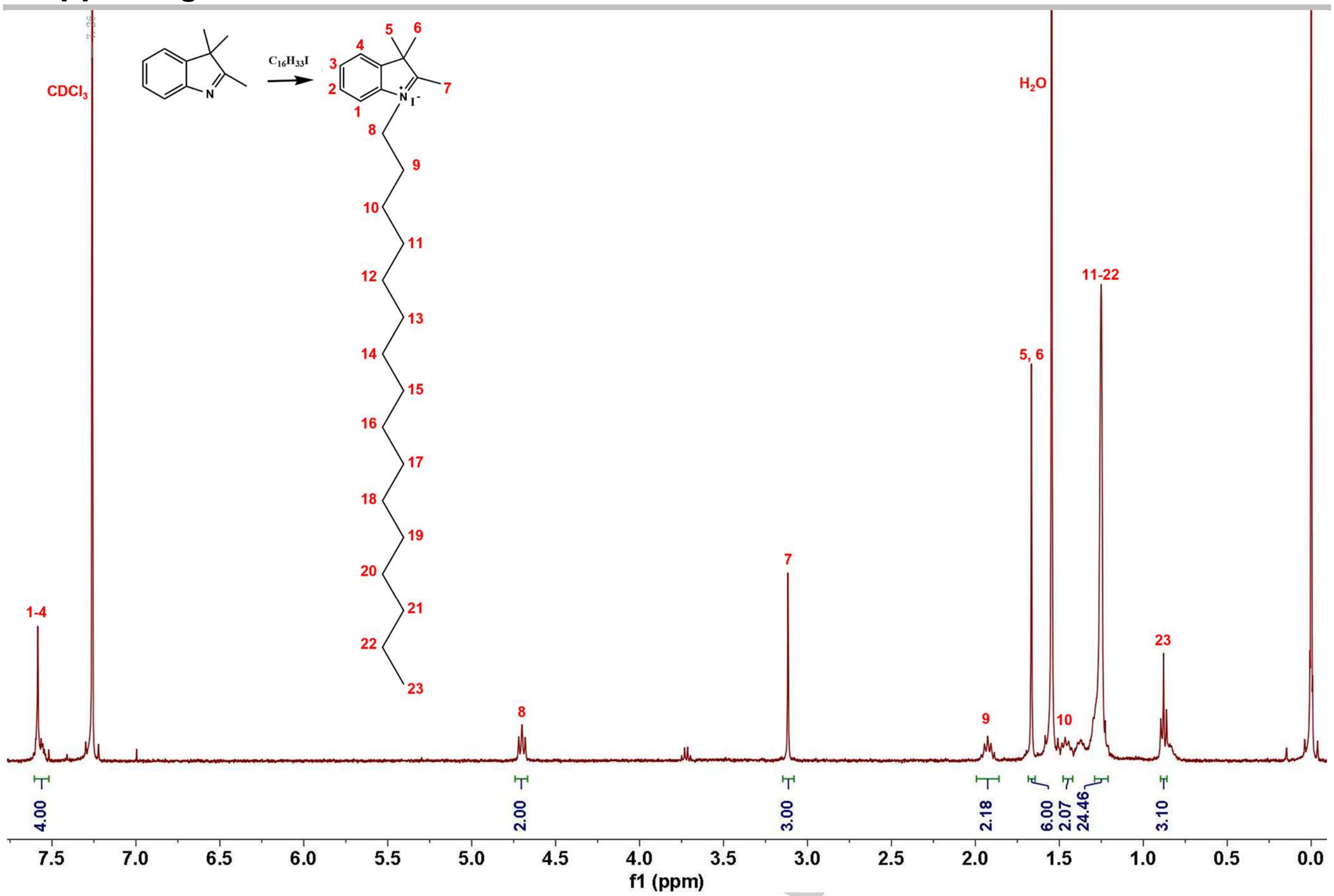

Figure S4. ^{1}H NMR spectrum of Compound 2 (400 MHz, 298K, $CDCl_3$).

WILEY

Supporting Information

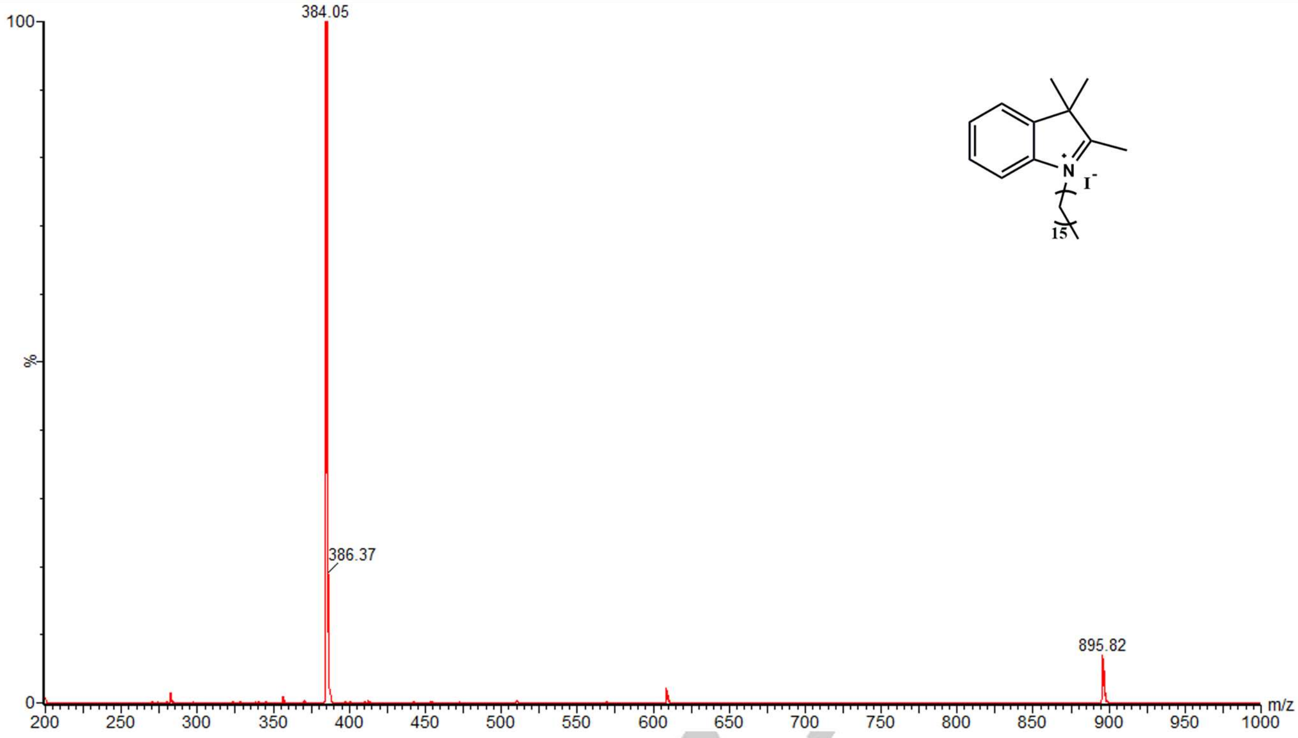

Figure S5. ESI-MS spectrum of Compound 2.

WILEY

Supporting Information

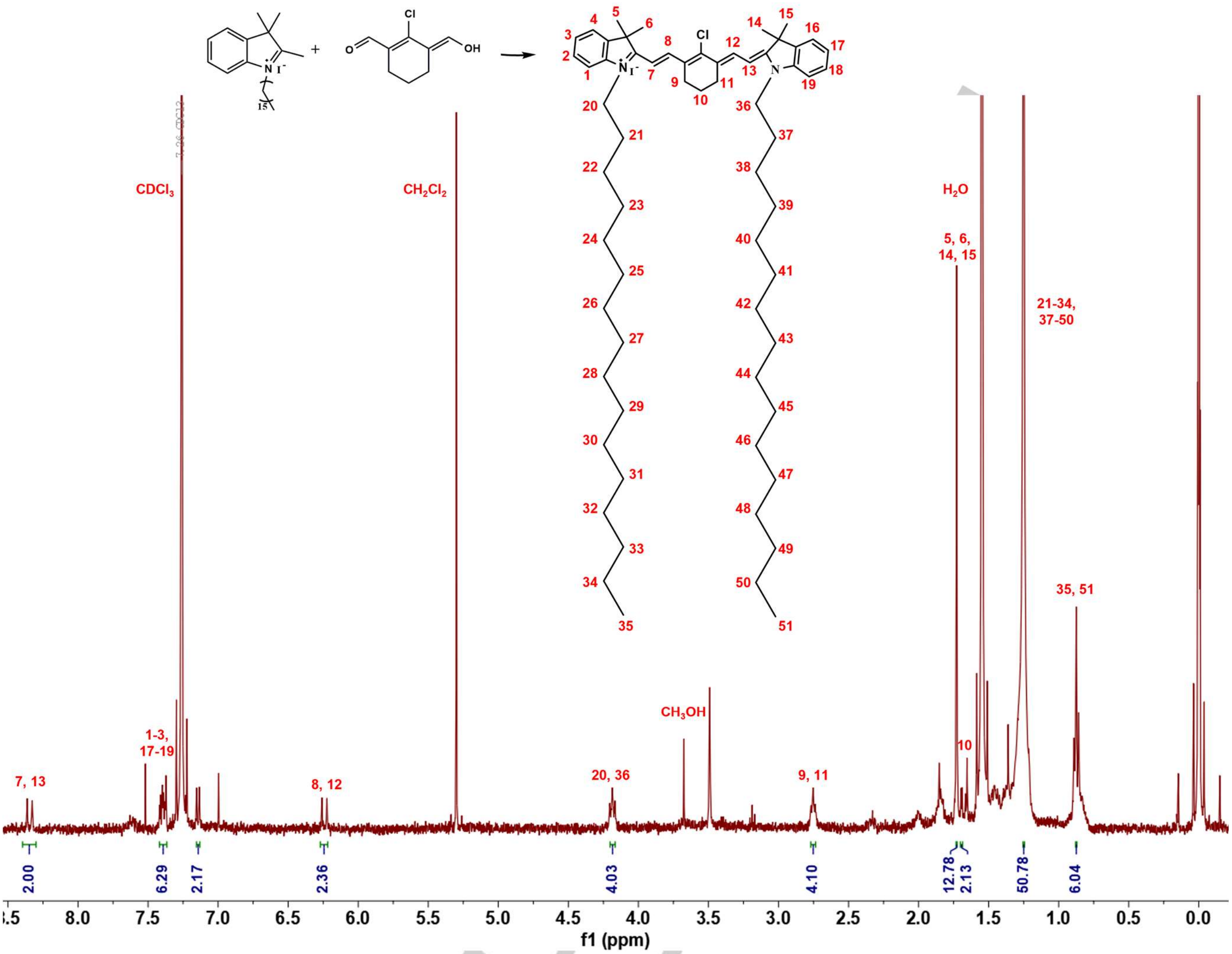

Figure S6. ^{1}H NMR spectrum of IR780-C16 (400 MHz, 298K, CDCl$_3$).

Supporting Information

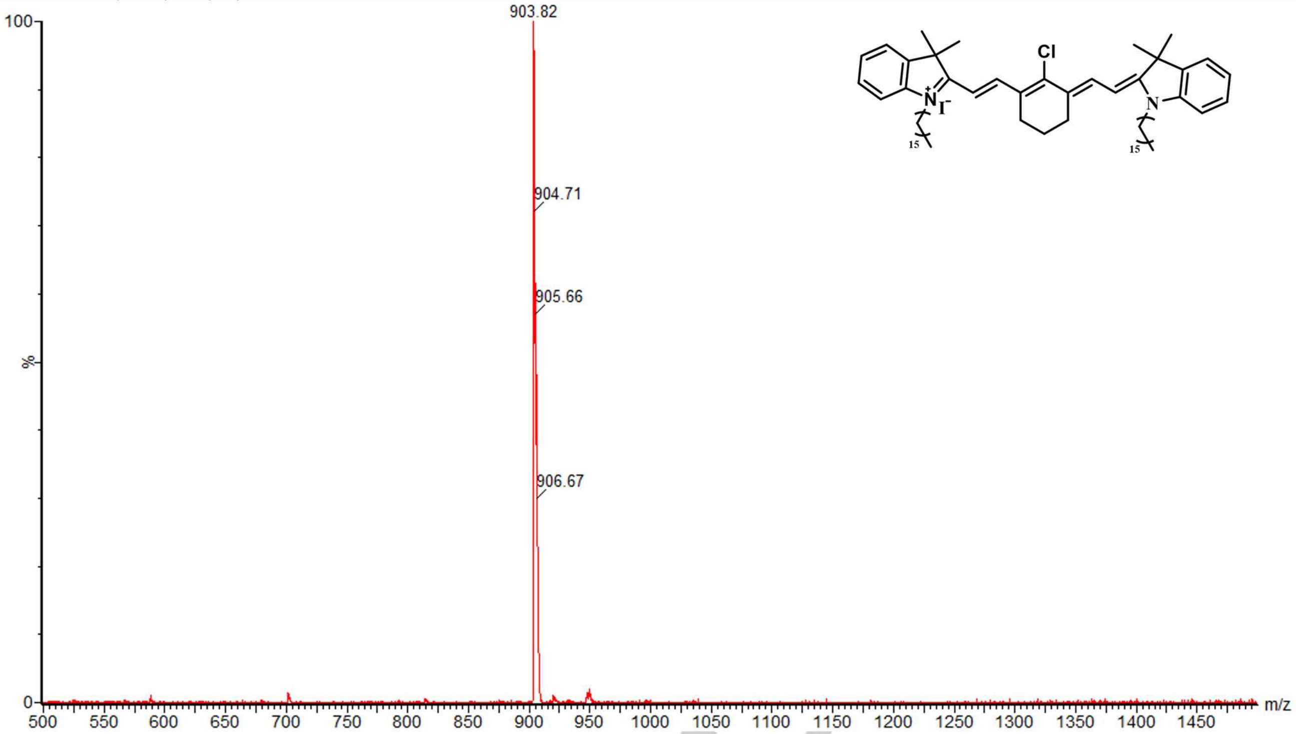

Figure S7. ESI-MS spectrum of IR780-C16.

WILEY

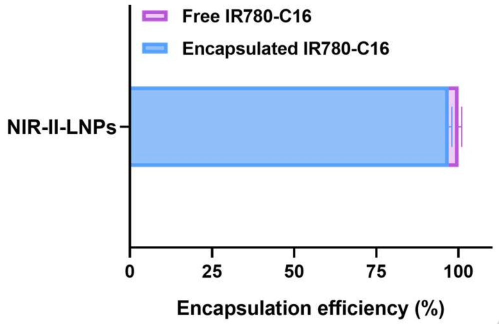

Figure S8. Encapsulation efficiency of IR780-C16 within NIR-II-LNPs (*n* = 3). Results are displayed as mean ± SD.

WILEY-VCH

Supporting Information

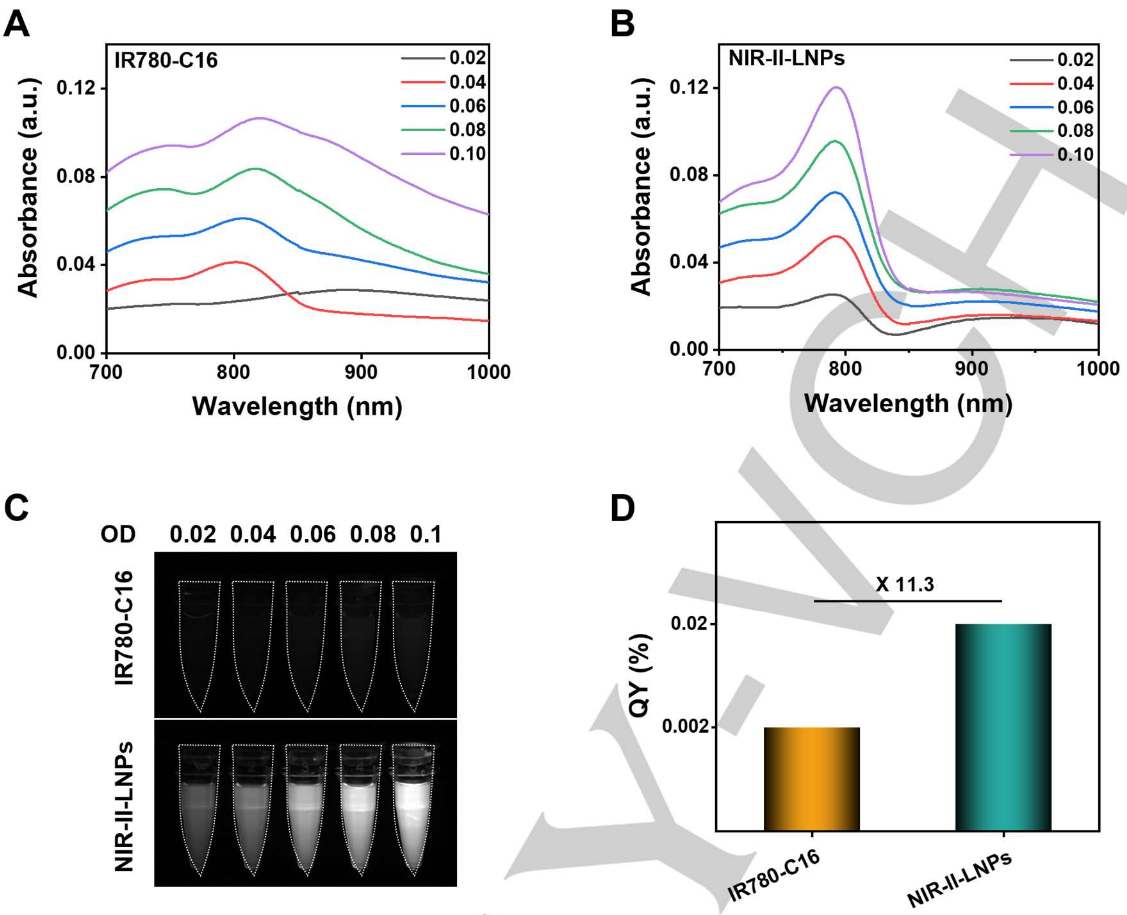

Figure S9. Comparison of NIR-II QYs (slope method) for IR780-C16 and NIR-II-LNPs. **(A, B)** Absorption spectra of five concentrations of **(A)** IR780-C16 and **(B)** NIR-II-LNPs in PBS. **(C)** The NIR-II imaging (> 1100 nm) of the above samples under 808 nm laser excitation. **(D)** The QYs of IR780-C16 and NIR-II-LNPs over 1100 nm.

WILEY-VCH

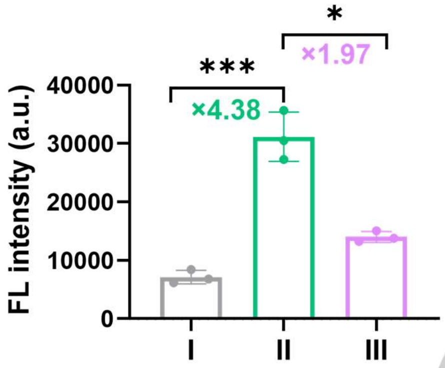

Figure S10. Quantification of fluorescent intensity of NIR-II-LNPs@mRNA in iLNs sections for Fig. 2F.

WILEY-VCH

Supporting Information

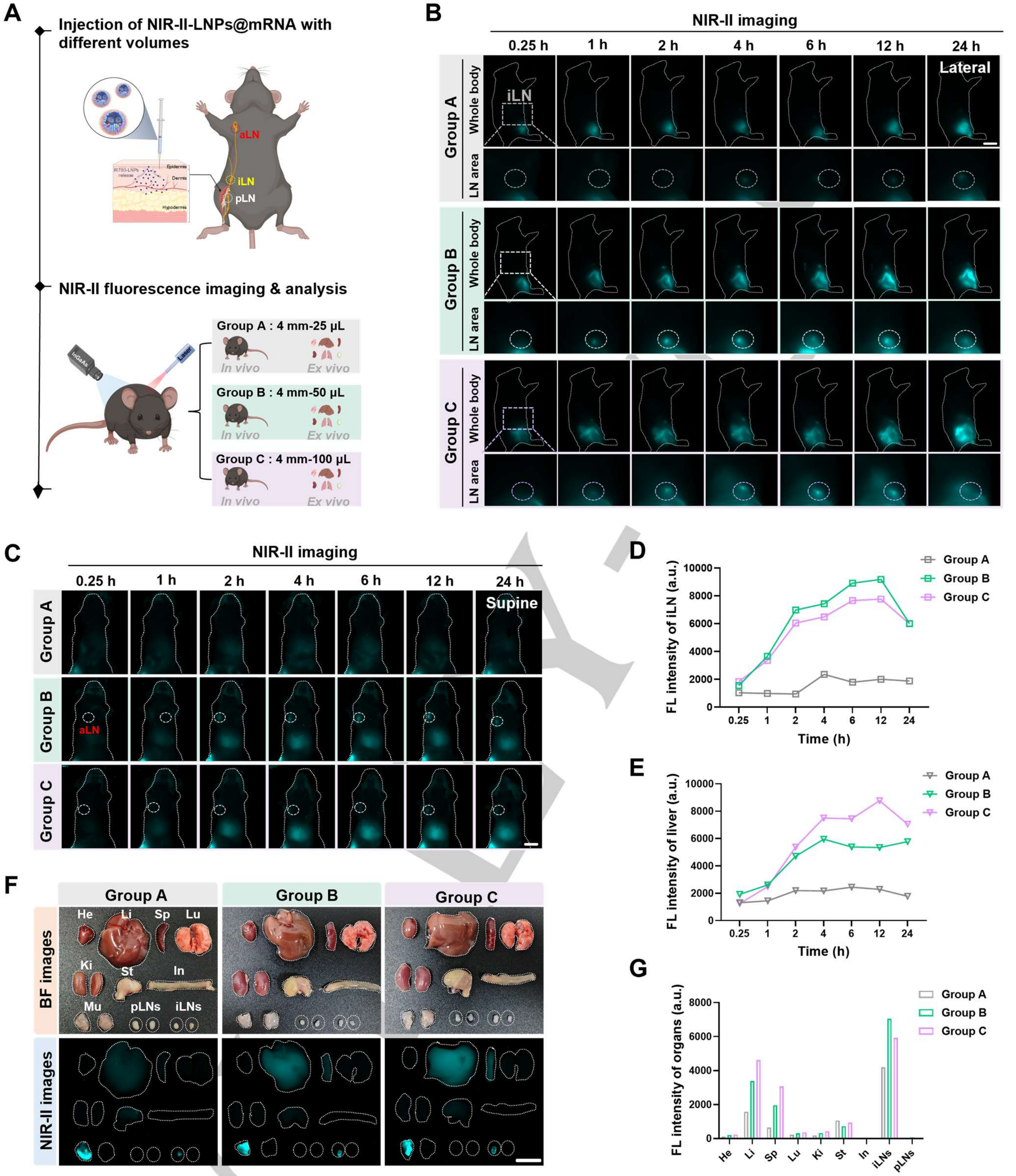

Figure S11. Influence of vaccine formulation volume on the kinetics of NIR-II-LNPs@mRNA transport to iLNs. **(A)** A schematic of the NIR-II imaging study with C57 mice receiving different injection protocols. **(B, C)** Whole-body and high-magnification fluorescent images of mice after intramuscular injection of NIR-II-LNPs@mRNA. Scale bars =1 cm. **(D, E)** Time-dependent changes of fluorescence intensity in iLNs **(D)** and liver **(E)** *in vivo*. **(F)** *EX vivo* fluorescence imaging of muscle, LNs, and major organs harvested from the mice at 24h post-injection. **(G)** Quantification of fluorescence intensity of excised muscle, LNs, and major organs.

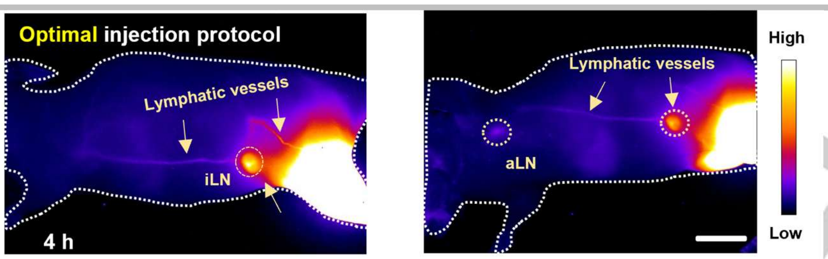

Figure S12. The NIR-II fluorescence imaging of lymphatics in mice after intramuscular injection of NIR-II-LNPs@mRNA. Scale bar, 1 cm.

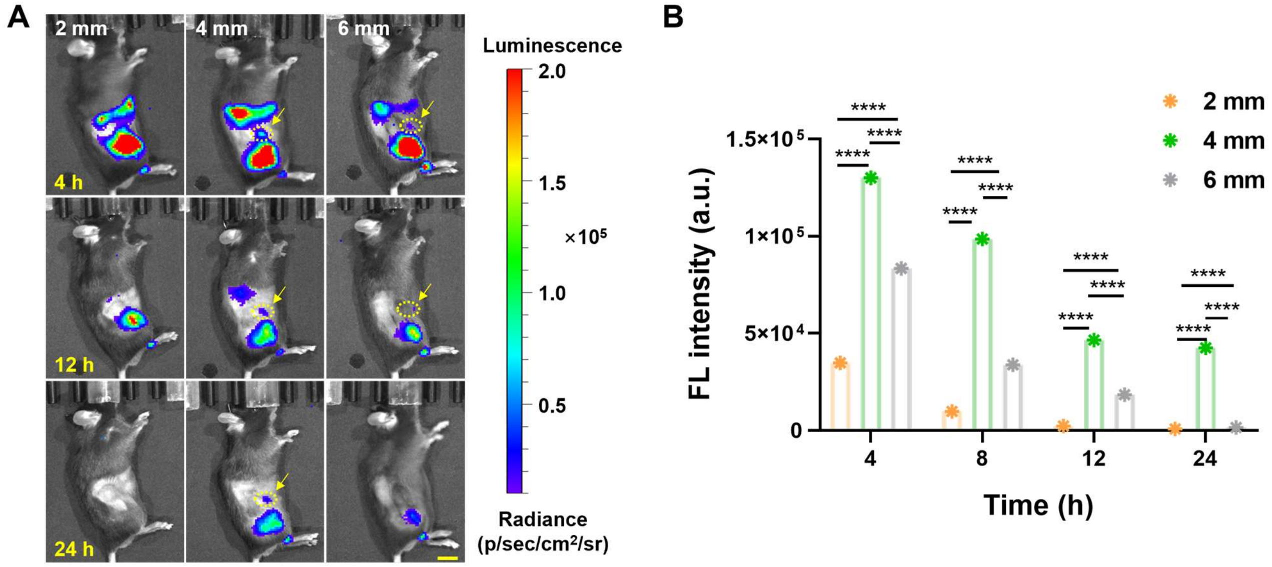

Figure S13. **(A)** *In vivo* bioluminescent images after NIR-II-LNPs@mRNA$^{OVA\text{-}Luc}$ injections at varying intramuscular depths and **(B)** quantified signal intensity (n = 3).

WILEY-VCH

Supporting Information

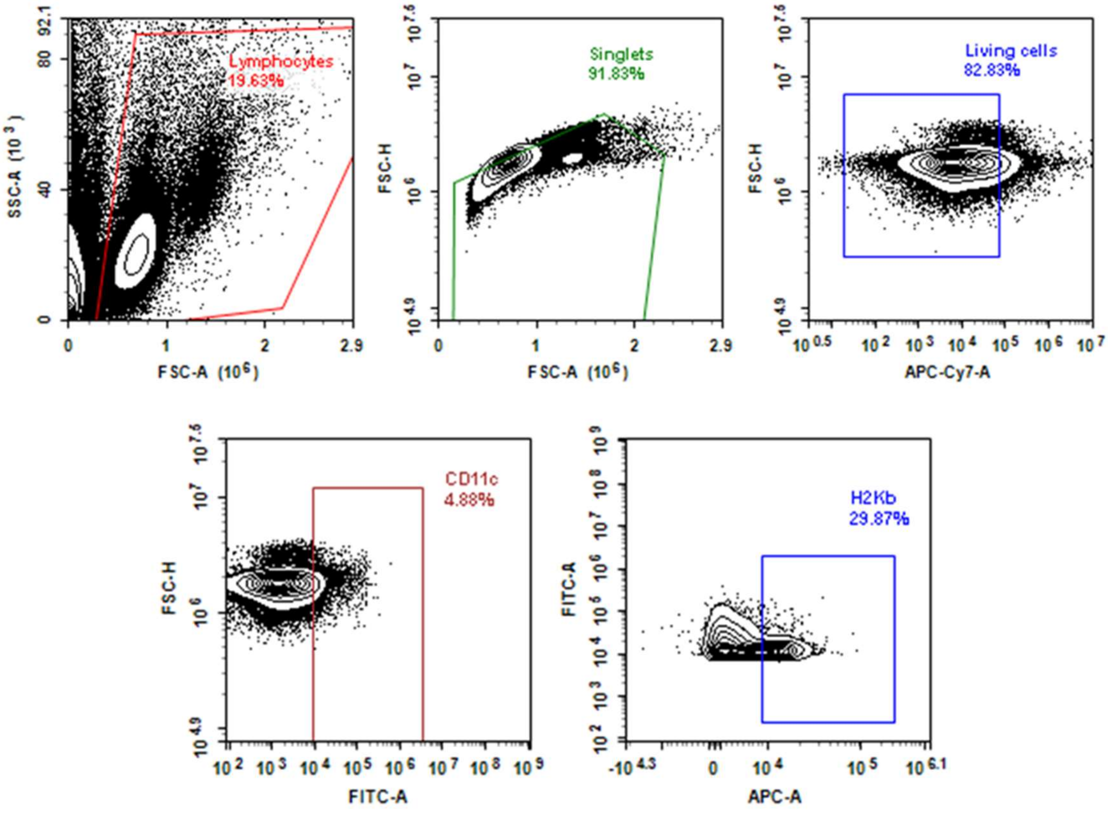

Figure S14. The gating strategy for the expression of OVA-H2Kb on DCs in the dLNs by flow cytometry.

WILEY

Supporting Information

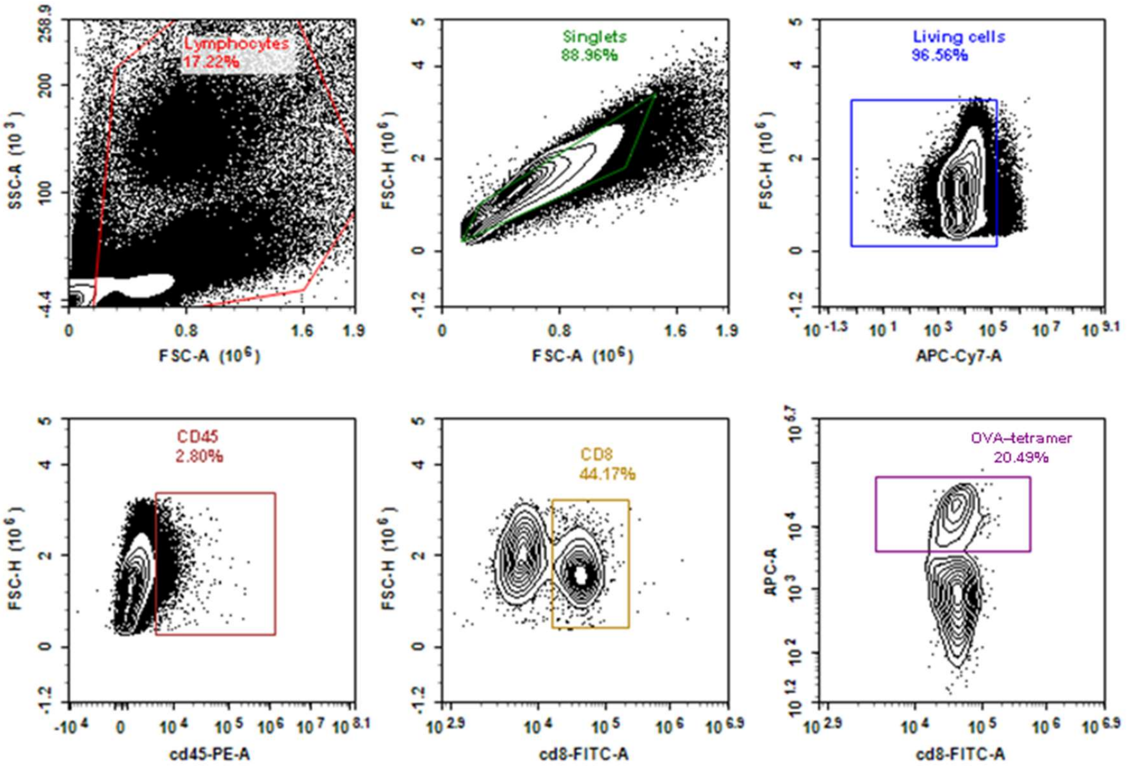

Figure S15. The gating strategy for the OVA-tetramer^{+}CD8^{+} T cells in peripheral blood by flow cytometry.

WILEY

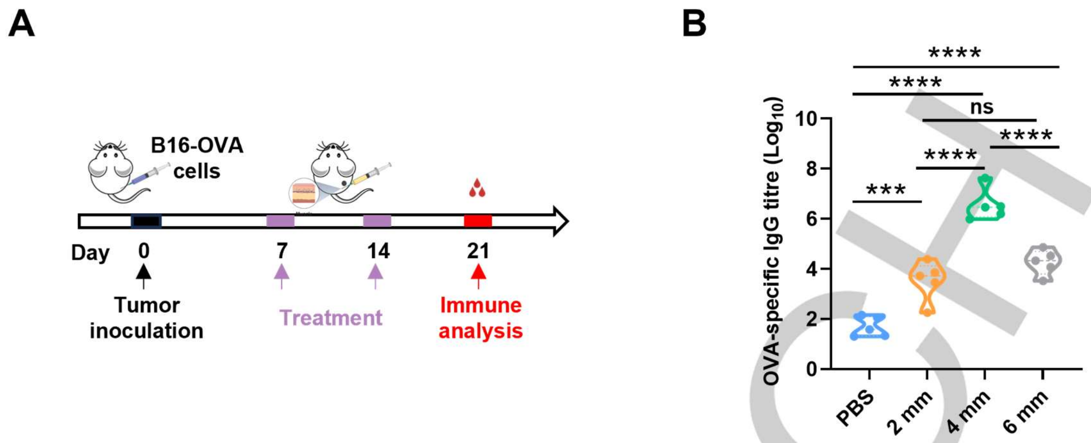

Figure S16. **(A)** Schematic of the experimental timeline for the treatment protocol in the B16F10-OVA mouse model. **(B)** ELISA of total OVA-specific IgG amounts in the serum of mice immunized with PBS or NIR-II-LNPs@mRNA^{OVA} at varying intramuscular depths (2 mm, 4 mm, 6 mm).

WILEY-VCH

Supporting Information

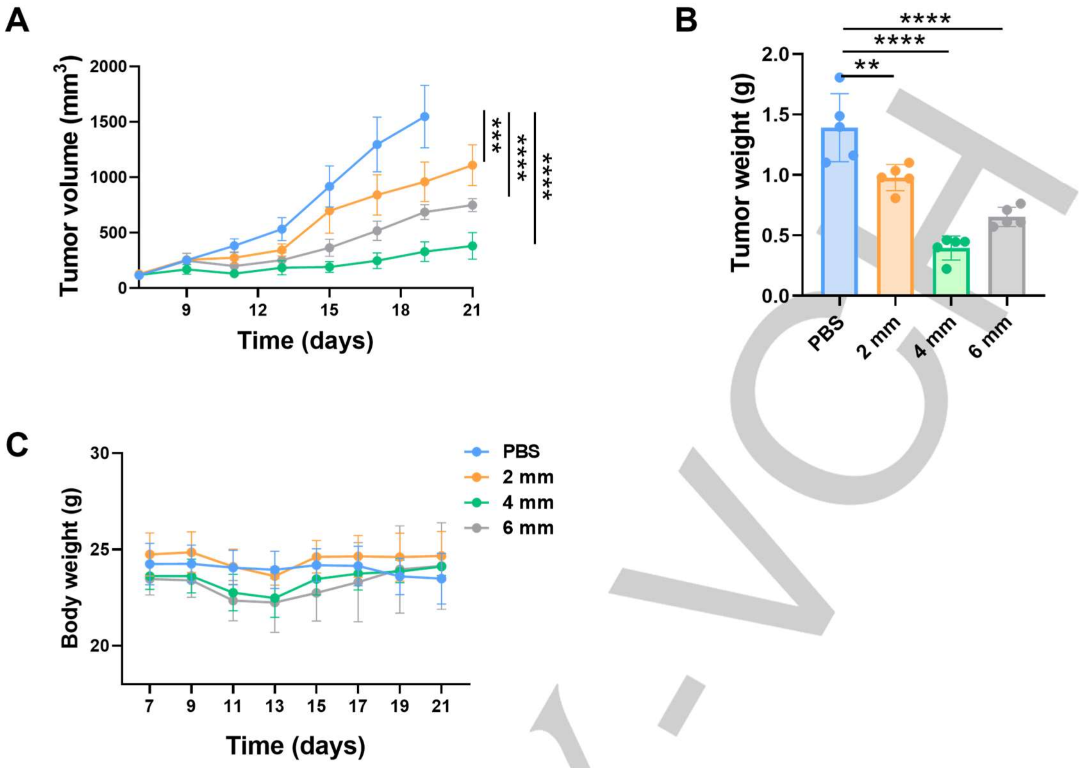

Figure S17. **(A)** Average tumor growth curves of mice receiving different treatments (*n* = 5/group). (B) Final tumor weights of the tumor-bearing mice treated with PBS or NIR-II-LNP@mRNA^{OVA} (5 μg mRNA per mouse) at varying intramuscular depths (2 mm, 4 mm, 6 mm). **(C)** Weight changes of the mice bearing B16F10-OVA tumors after different treatments.

WILEY-VCH

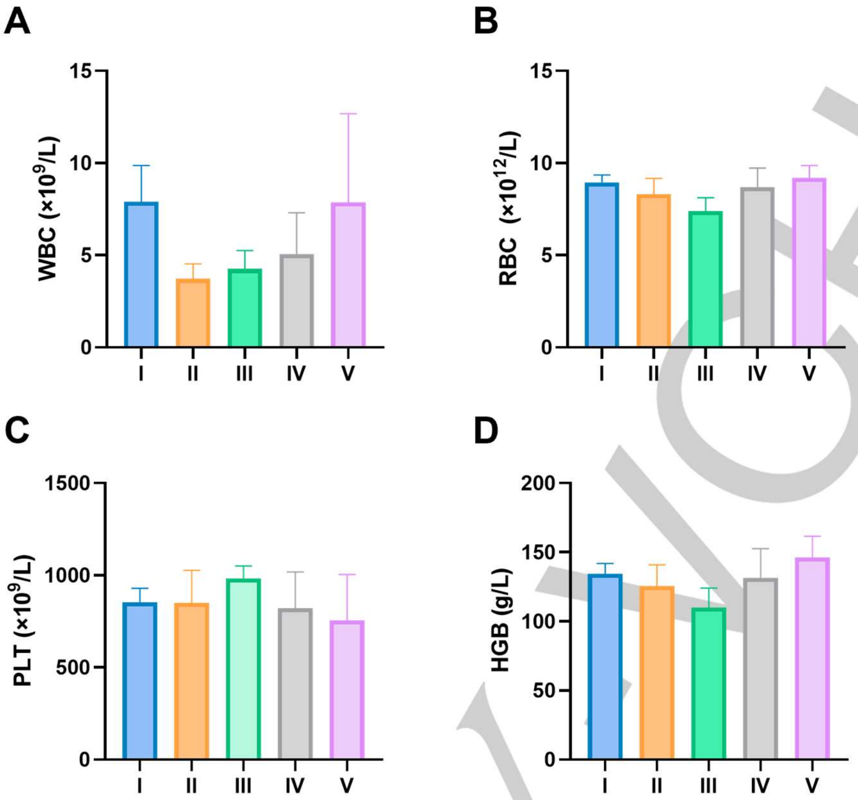

Figure S18. Blood safety of NIR-II-LNPs@mRNA^{E7} with different injection regimens (5 μg mRNA per mouse). **(A)** White blood cell (WBC). **(B)** Red blood cell (RBC). **(C)** Platelet (PLT) and **(D)** Hemoglobin (HGB) levels in the mice after various treatments. Each group consisted of 3 mice. Error bars were based on SD. I, PBS; II, 2 mm-25 μL; III, 4 mm-25 μL; IV, 6 mm-25 μL; V, 4 mm-50 μL.

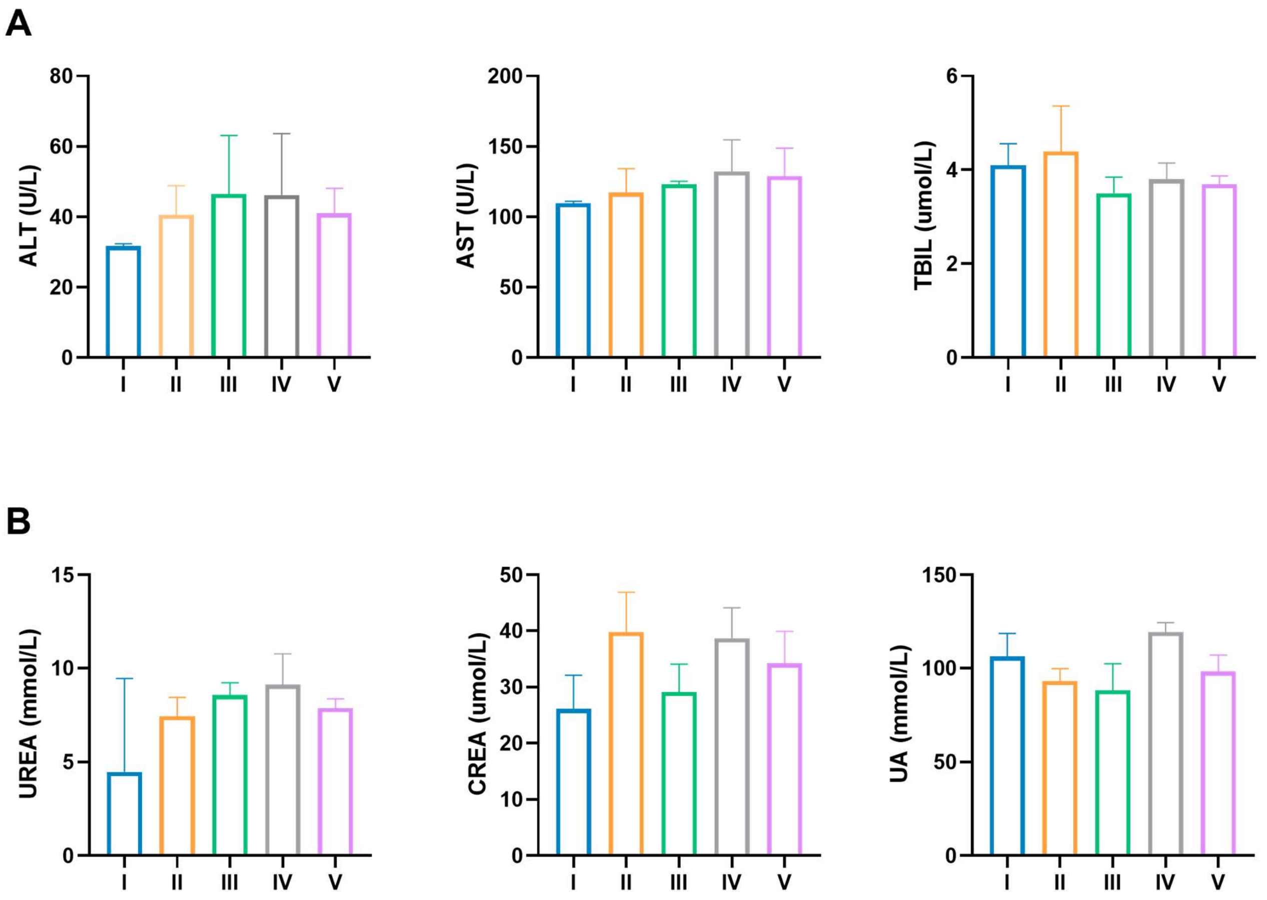

Figure S19. **(A)** Hepatic functional indexes. **(B)** Renal function indices of mice after various treatments. Each group has 3 mice. Mean ± SD. I, PBS; II, 2 mm-25 μL; III, 4 mm-25 μL; IV, 6 mm-25 μL; V, 4 mm-50 μL (NIR-II-LNP@mRNA^{E7}, 5 μg mRNA per mouse).

WILEY

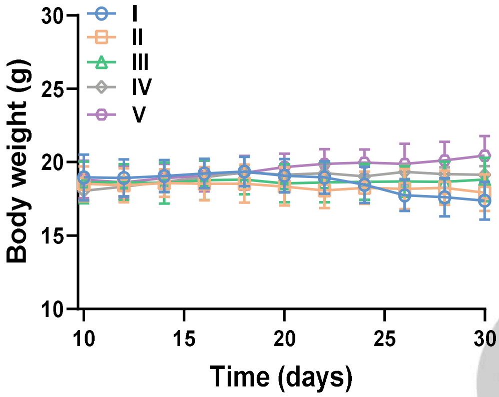

Figure S20. The body-weight changes of mice in various groups. I, PBS; II, 2 mm-25 μL; III, 4 mm-25 μL; IV, 6 mm-25 μL; V, 4 mm-50 μL (NIR-II-LNP@mRNA^{E7}, 5 μg mRNA per mouse).

WILEY-VCH

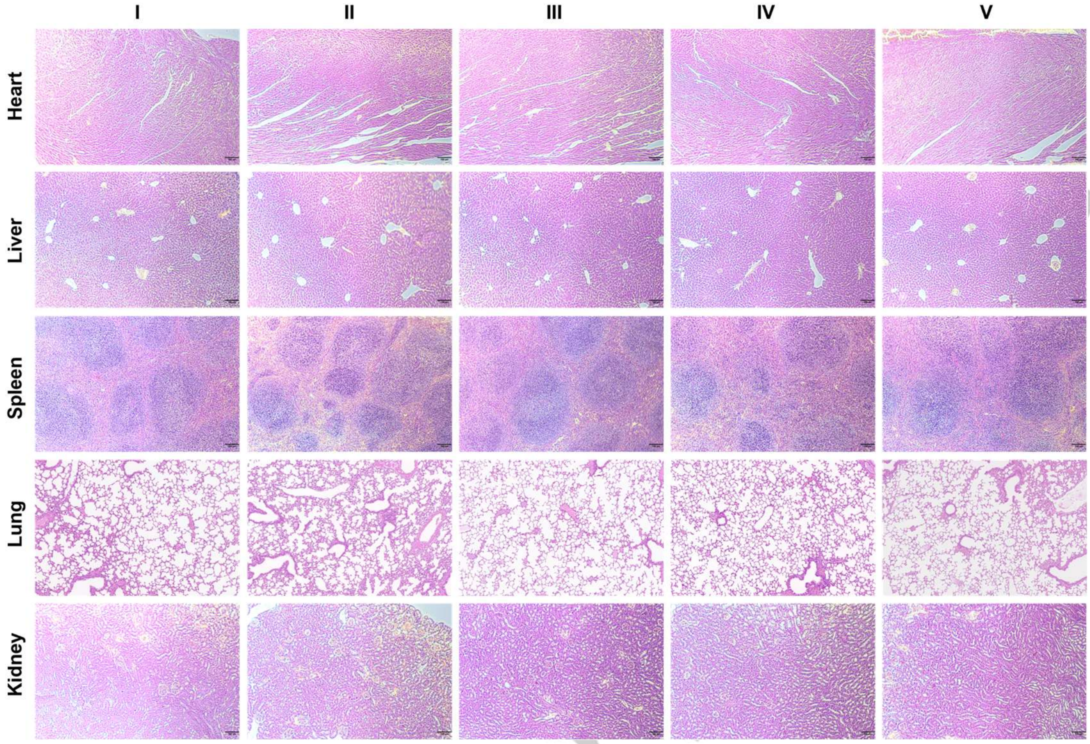

Figure S21. H&E staining of main organs from the TC-1 subcutaneous tumor-bearing mice after different treatments. Scale bar, 100 μm. The body-weight changes of mice in various groups. I, PBS; II, 2 mm-25 μL; III, 4 mm-25 μL; IV, 6 mm-25 μL; V, 4 mm-50 μL (NIR-II-LNP@mRNA^{E7}, 5 μg mRNA per mouse).

WILEY

Supporting Information

Table S1 The diameter of the TC-1 tumors in indicated groups.

Sample	Length (cm)	Width (cm)	Group	Sample	Length (cm)	Width (cm)	Group
**1**	2.3	1.3		**16**	1.0	1.3	
**2**	1.2	1.7		**17**	0.8	1.2	
**3**	1.8	1.4	**I**	**18**	1.3	0.9	**IV**
**4**	1.7	1.2		**19**	1.1	1.0	
5	1.4	1.4		**20**	1.3	0.9	
**6**	1.3	1.4		**21**	0.7	0.9	
**7**	1.2	1.4		**22**	0.6	0.7	
**8**	1.2	1.6	**II**	**23**	1.1	0.8	**V**
**9**	1.1	1.5		**24**	0.9	0.9	
**10**	1.4	1.2		**25**	0.9	0.8	
**11**	0.8	0.9					
**12**	0.9	0.9					
**13**	1.2	0.8	**III**				
**14**	1.0	1.1					
**15**	1.1	1.1					

WILEY-VCH
